# Pancreatic Neuroendocrine Tumors: Molecular Mechanisms and Therapeutic Targets

**DOI:** 10.3390/cancers13205117

**Published:** 2021-10-12

**Authors:** Chandra K. Maharjan, Po Hien Ear, Catherine G. Tran, James R. Howe, Chandrikha Chandrasekharan, Dawn E. Quelle

**Affiliations:** 1 Department of Neuroscience and Pharmacology, Carver College of Medicine, University of Iowa, Iowa City, IA 52242, USA; cmaharjan@ufl.edu; 2 Department of Surgery, Carver College of Medicine, University of Iowa, Iowa City, IA 52242, USA; pohien-ear@uiowa.edu (P.H.E.); catherine-tran@uiowa.edu (C.G.T.); james-howe@uiowa.edu (J.R.H.); 3 Department of Internal Medicine, Carver College of Medicine, University of Iowa, Iowa City, IA 52242, USA; chandrikha-chandrasekharan@uiowa.edu; 4 Department of Pathology, Carver College of Medicine, University of Iowa, Iowa City, IA 52242, USA; 5 Holden Comprehensive Cancer Center, University of Iowa, Iowa City, IA 52242, USA

**Keywords:** pancreatic neuroendocrine tumors, molecular mechanisms, pNET models

## Abstract

**Simple Summary:**

Pancreatic neuroendocrine tumors (pNETs) are rare, indolent cancers whose causation is only partly understood. An increasing number of studies have uncovered molecular changes associated with pNETs, helping to identify common disease mechanisms. This knowledge has guided current pNET therapies that can effectively slow progression of the disease. However, tumors often become resistant to available therapies, necessitating a deeper understanding of mechanisms driving disease progression in order to develop new treatments. Here, we provide a comprehensive review of pNET-associated molecular alterations and existing pNET models to illustrate potential areas for advancement in research and therapy.

**Abstract:**

Pancreatic neuroendocrine tumors (pNETs) are unique, slow-growing malignancies whose molecular pathogenesis is incompletely understood. With rising incidence of pNETs over the last four decades, larger and more comprehensive ‘omic’ analyses of patient tumors have led to a clearer picture of the pNET genomic landscape and transcriptional profiles for both primary and metastatic lesions. In pNET patients with advanced disease, those insights have guided the use of targeted therapies that inhibit activated mTOR and receptor tyrosine kinase (RTK) pathways or stimulate somatostatin receptor signaling. Such treatments have significantly benefited patients, but intrinsic or acquired drug resistance in the tumors remains a major problem that leaves few to no effective treatment options for advanced cases. This demands a better understanding of essential molecular and biological events underlying pNET growth, metastasis, and drug resistance. This review examines the known molecular alterations associated with pNET pathogenesis, identifying which changes may be drivers of the disease and, as such, relevant therapeutic targets. We also highlight areas that warrant further investigation at the biological level and discuss available model systems for pNET research. The paucity of pNET models has hampered research efforts over the years, although recently developed cell line, animal, patient-derived xenograft, and patient-derived organoid models have significantly expanded the available platforms for pNET investigations. Advancements in pNET research and understanding are expected to guide improved patient treatments.

## 1. pNET Introduction, Pathological Features and Classification

The incidence and prevalence of NETs have risen more than 6-fold in the United States over the past 40 years, largely reflecting an increase in the diagnosis of early stage disease [1]. NETs are a heterogenous group of slowly growing neoplasms arising in the neuroendocrine tissues of mainly the gastrointestinal (GI) tract (small intestine, appendix, and large intestine), lungs, and pancreas. Primary NETs can also develop in the thyroid, adrenal, pituitary glands, and ovaries. Notably, some NETs, particularly those in the small bowel, display a spectrum of symptoms (called carcinoid syndrome) that include flushing, diarrhea, and bronchospasm, which are associated with tumoral hypersecretion of vasoactive amines, such as serotonin and histamine [2].

Pancreatic NETs (pNETs) constitute only 1–2% of all pancreatic neoplasms [3]. Although these originate primarily from aberrantly proliferating cells of the endocrine pancreas, they can also develop from pluripotent cells of the exocrine pancreas [4,5]. The annual incidence of pNETs is less than 1 per 100,000 individuals; however, their rate has significantly increased over the past few decades [1]. Most pNETs occur sporadically, but some occur in association with hereditary multi-tumor predisposition syndromes, such as multiple endocrine neoplasia type 1 (MEN1), von-Hippel-Lindau (VHL), neurofibromatosis type 1 (NF-1), tuberous sclerosis complex (TSC), and Cowden syndrome (CS) [6]. Based on symptoms manifested by tumor-secreted hormones, pNETs are categorized as functional or non-functional. Insulinoma, gastrinoma, and rare pNET subtypes such as glucagonoma, VIP (vasoactive intestinal peptide)-oma, and somatostatinoma are some examples of functional pNETs, which manifest hormone-related clinical symptoms that may help in diagnosing these tumors at an earlier stage [4,7]. In contrast, non-functional pNETs, which represent 60–90% of all pNETs, are often asymptomatic and likely to remain undiagnosed until they are advanced and unresectable. As such, patients with non-functional pNETs tend to have worse outcomes compared to those with functional tumors [2,4].

The 2019 World Health Organization (WHO) tumor grading system defines pNETs as well-differentiated (WD) pancreatic neuroendocrine neoplasms (pNENs) and hence, a pNEN subset [8]. The second subset comprises pancreatic neuroendocrine carcinomas (pNECs), which are poorly differentiated pNENs. Mixed neuroendocrine-non-neuroendocrine neoplasms (miNENs) are grouped as the third pNEN subset to represent rare, poorly defined pancreatic tumors possessing specific molecular and pathological features of neuroendocrine and non-neuroendocrine components [9].

A pNET cell displays minimal to moderate atypia, lacks necrosis, and exhibits intense staining of NE differentiation markers, synaptophysin and chromogranin [10]. In contrast, a pNEC consists of highly atypical cells of varying sizes and exhibits faint staining of synaptophysin and chromogranin A. pNETs are further subdivided into grade 1 (G1), grade 2 (G2), and grade 3 (G3) based on increasing order of their proliferation index (Ki-67 index <3, 3–20, and >20, for G1, G2, and G3 pNETs, respectively), whereas pNECs are invariably G3. Common alterations in pNECs, such as genetic mutations in the *RB1* and *TP53* genes, are distinct from those of pNETs (e.g., *MEN1*, *ATRX*, *DAXX*) suggesting pNECs originate *de novo* and not via pNET dedifferentiation [11,12]. miNENs could be well or poorly differentiated and usually possess an aggressive phenotype by virtue of its NEC component [8,9]. On the other hand, they mimic adenocarcinomas histologically and at the molecular level by exhibiting high rates of *TP53*, *BRAF*, and *KRAS* mutations.

To supplement the WHO classification system with additional criteria refining prognostic stratification and therapeutic decision-making for pNENs, the European Neuroendocrine Tumor Society (ENETS) and American Joint Committee on Cancer/Union for International Cancer Control (AJCC/UICC) introduced the TNM-staging system [13,14]. ‘T’ stages (1–4) describe tumor size and invasion into the surrounding tissues and blood vessels; ‘N’ indicates the presence of regional lymph node metastases; ‘M’ indicates the presence of distant metastases [3,13,14]. Accurate grading and staging of pNENs guides prognostication, choice of treatment modality, and clinical decision making [15]. Interestingly, a recent multicenter retrospective study of pNEN susceptibility identified gender differences in age at diagnosis, associated co-morbidities (such as type II diabetes), and potential risk factors [16]. Although females were slightly more likely to be diagnosed at a younger age compared to males, in females the presence of type II diabetes was associated with higher tumor grade and metastatic disease. The authors suggest a gender-tailored approach could be important in improving pNEN clinical management.

There are several effective therapies against pNETs that have improved patient outcomes, although surgical resection is the only curative treatment for localized tumors [15]. Approximately 40% of patients present with advanced, metastatic disease [1], requiring systemic pharmacological or radiation-based therapies. The Food and Drug Administration (FDA) approved drug therapies used in the management of advanced, unresectable or metastatic pNETs include somatostatin analogs such as octreotide LAR (long acting repeat) and lanreotide; mTOR inhibitor, everolimus; multitargeted tyrosine kinase inhibitor, sunitinib; and chemotherapeutic agents, 5 fluorouracil, and streptozotocin. Although the combination of 5 fluorouracil and streptozotocin is approved, the capecitabine and temozolamide combination is often used in clinical practice for their better side effect profile and more reliable efficacy. Peptide receptor radionuclide therapy (PRRT) using radiolabeled somatostatin analogue, ^177^Lu-Dotatate (Lutathera), is also FDA approved for the management of advanced pNETs [17,18]. For pNET patients with carcinoid syndrome (less than 1%) [19], telotristat ethyl is an oral tryptophan hydroxylase 1 (TPH1) inhibitor that can provide relief from symptoms, particularly by decreasing the number of daily bowel movements [20]. Together, the broadly relevant therapies described above and context dependent use of telotristat ethyl have improved outcomes and quality of life of pNET patients.

Despite these advances, a significant percentage of pNET patients are non-responsive to existing anti-tumor therapies or they develop acquired drug resistance. This highlights an urgent need to develop more effective therapies, which can only be achieved through a better understanding of the molecular mechanisms driving pNET pathogenesis.

**Figure 1:**
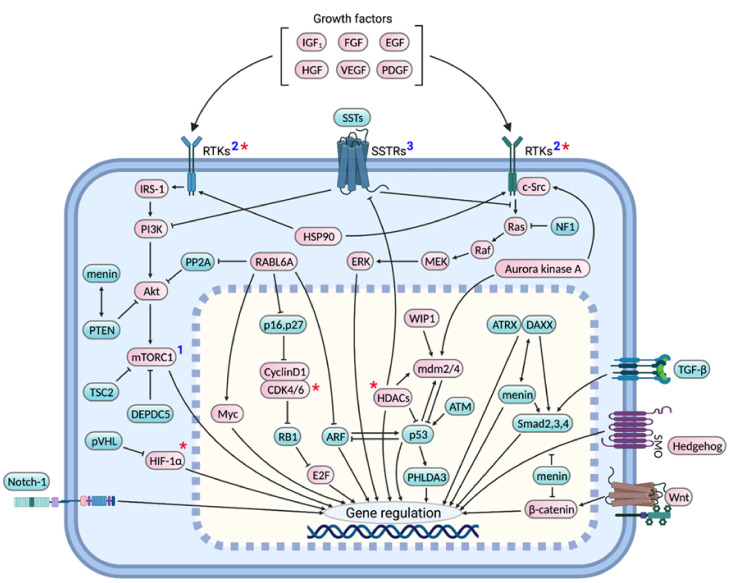
Diagrammatic illustration of important signaling proteins and pathways that drive pNET cell survival, proliferation, angiogenesis, and metastasis. Proteins having oncogenic roles in pNETs are shown in pink boxes whereas pNET suppressors are shown in green. Numbers highlighted in blue indicate FDA approved drugs for therapy that target the corresponding proteins: 1-everolimus, 2-sunitinib, and 3-somatostatin analogues. Asterisks in red denote additional targets being evaluated for pNET therapy. Arrows, activating events; perpendicular bars, inhibitory events. Schematic developed using BioRender software (Toronto, Canada).

Several molecular profiling studies have revealed important pNET-signature genes and pathways. However, which molecular alterations are essential for pNET genesis and progression is not clearly understood. Our review provides a comprehensive discussion of: (i) genes and signaling pathways shown to be frequently altered in pNETs by molecular profiling studies, as well as their prognostic and therapeutic significance, (ii) genes associated with familial pNETs with a focus on the downstream mechanisms underlying pNET development, (iii) additional proteins and signaling pathways, whose genes or expression might remain unchanged yet whose altered activities are functionally essential for pNET development and progression, (iv) interplay between these various signaling pathways, (v) preclinical and clinical studies evaluating pNET targeted therapies, and (vi) in vitro and in vivo pNET models used for biological studies and drug screenings. Figure 1 provides a consolidated diagram of important signaling pathways discussed herein whose dysregulation drives pNET pathogenesis.

## 2. pNET-Associated Genes and Signaling Pathways

**Figure 2:**
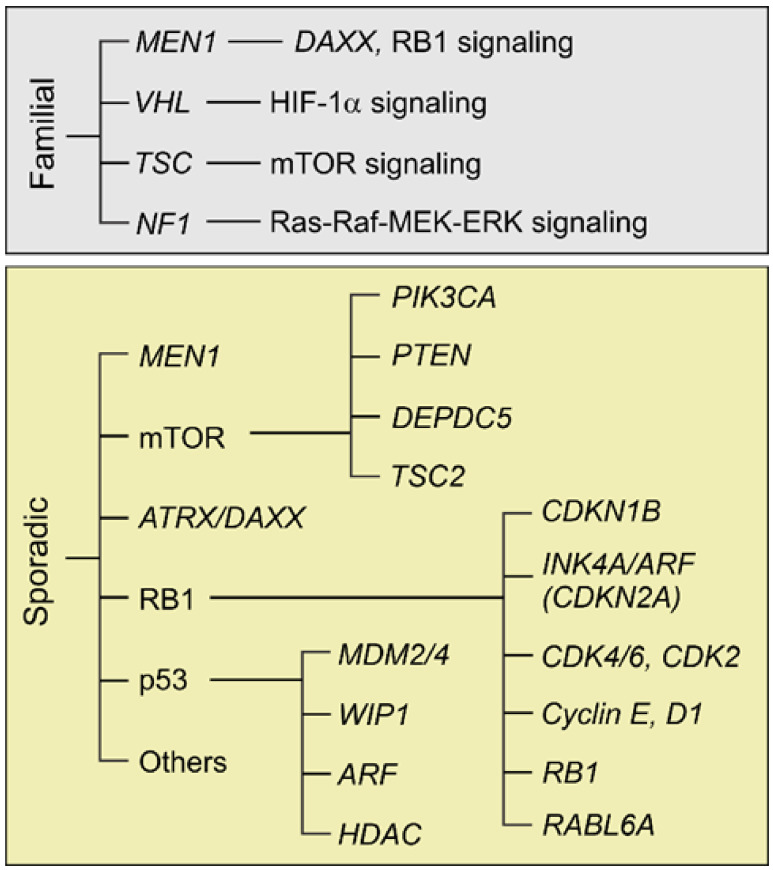
Schematic of frequently altered genes and pathways in familial and sporadic pNETs. Genes (italics) and pathways (non-italics).

The molecular pathogenesis of pNETs is only partially understood. By comparison, much more is known about pancreatic ductal adenocarcinomas (PDACs), the most common type of pancreatic cancer, which arise from exocrine cells and are primarily driven by KRAS activation [21] and other major genetic changes, including *TP53* mutation [22]. Several groups have conducted whole genome and exome sequencing of patient-derived pNETs to gain more insight into genetic alterations driving their development. One of the major conclusions has been that pNETs, unlike PDACs, have a low mutational burden [23,24]. These and other molecular profiling studies, along with functional investigations performed in pNET cells and mouse models, have identified the most frequently altered, biologically relevant genes and pathways underlying the pathogenesis of familial and sporadic pNETs (Figure 2).

### 2.1. Menin

The *MEN1* gene encodes a 610-amino acid nuclear scaffold protein called menin, which plays an important role in chromosomal remodeling and gene transcription. Inheritance of germline mutations in one *MEN1* allele from either parent leads to a familial autosomal dominant tumor syndrome called multiple endocrine neoplasia type 1 (MEN 1). Of all the different familial causes of pNETs, MEN1 syndrome remains the most frequent. Historically, linkage analysis and loss of heterozygosity analysis of tumors from affected individuals mapped the gene to the chromosomal region 11q13 [25,26], which led to positional cloning studies identifying the *MEN1* gene [27,28]. Several studies suggest that oncogenesis in MEN1 individuals involves unmasking of the mutated *MEN1* allele at the disease locus by loss of the remaining wild-type copy, in agreement with Knudson’s two-hit mutation model for tumor suppressor genes originally proposed for *RB1* loss in retinoblastoma [29,30,31].

Over 90% of MEN1 patients exhibit one or more different types of endocrine tumors by the age of 50 [6]. This mainly includes parathyroid (with a frequency of 95–100%), pancreatic (80–100%), and pituitary (54–64%) tumors [6,32,33,34,35]. Nearly all MEN1 patients (80–100%) develop NF-pNETs, which remain small and asymptomatic in most individuals. Insulinomas are the most common functional pNETs in MEN1, observed in 18% of MEN1 patients [6]. While more than half of MEN1 individuals also develop gastrinomas, the vast majority (greater than 80%) are duodenal, and only a small percentage of these tumors are pancreatic. Glucagonomas (3%), VIPomas (3%), GRF (growth hormone-releasing factor)-omas (3%), and somatostatinomas (<1%) are other less frequent functional pNETs associated with MEN1 [6].

In addition to its prominent role in familial pNETs, somatic *MEN1* mutations are arguably the most frequent genetic event found in sporadic pNETs (Table 1). Whole exome sequencing revealed *MEN1* somatic mutations are present in 40–56% of sporadic pNETs, far exceeding the incidence of alterations in any other single gene within these tumors [36,37,38,39]. This finding builds upon earlier studies showing *MEN1* mutations in 5–30% of patient pNETs [29,30,32,36,37]. Notably, several allelotyping and loss of heterozygosity (LOH) analyses have found allelic loss of *MEN1* or the chromosomal segment encompassing the gene on 11q13 in 30–70% of sporadic pNETs [31,33,34,35,38,39]. Since allelic deletions of *MEN1* may be 2–3 times more common than *MEN1* gene mutations, it has been suggested that other tumor suppressor genes on 11q13 may factor into tumorigenesis of these neoplasms [36]. On the other hand, *MEN1* mutations are usually co-incident with deletion of the other functional allele, ultimately leading to complete loss of menin activity [40,41,42]. Such results suggest that pathogenesis of non-familial, sporadic pNETs may recapitulate tumor development seen in MEN1 patients.

Exactly how menin suppresses pNET development is still unfolding although it clearly plays a critical role in the nucleus. Menin is a 68 kilodalton (kDa) protein that contains two carboxy-terminal nuclear localization sequences (NLS) and normally resides in the nucleus of all pancreatic cells [41,44,45]. Tumor-associated frameshift and nonsense mutations in *MEN1* encode truncated forms of the protein that lack one or both NLS and protein-protein interaction domains [30,44]. This correlates with abnormally high cytoplasmic expression of menin in the majority (80%) of sporadic pNETs, whereas the wild-type protein is almost exclusively nuclear in normal islets [30]. Interestingly, cytoplasmic mis-localization of menin is not dependent on *MEN1* mutation, possibly reflecting loss of nuclear interactions, unmasking of a functional nuclear export signal (NES) in menin, and/or altered localization of its partners that may physically mobilize menin into the cytoplasm [30].

In the nucleus, menin interacts with a variety of proteins involved in transcription and DNA damage repair. Its partners include JunD, ASK, FANCD2, Smad3, Pem, Nm23, NF-kB, and replication protein A (RPA2) [30]. Menin either inhibits the activities of its oncogenic partners or promotes the function of its tumor suppressive partners. For example, menin association with JunD and NF-kB proteins (p50, p52 and p65) blocks their transcriptional activities [46,47], whereas its binding and activation of Smad3 promotes growth inhibitory signaling by transforming growth factor β (TGFβ) [42]. FANCD2 is a DNA repair protein in the BRCA1 DNA repair pathway that is mutated in patients with Fanconi anemia, an inherited cancer-prone syndrome. Jin et al., showed that menin forms complexes with FANCD2, which are enhanced by gamma irradiation and associated with reduced DNA damage [48]. These data suggest menin helps repair damaged DNA and maintain genomic stability, in agreement with prior studies showing increased chromosomal breaks and instability in *MEN1* mutant pancreatic tumors [49].

Additional studies revealed that menin is a member of histone methyltransferase complex that promotes histone methylation [50] as well as gene expression of cyclin dependent kinase inhibitors, p27^KIP1^ and p18^INK4C^, to suppress pancreatic islet growth [51,52]. In islet cells, loss of *MEN1* enhances proliferation by accelerating S-phase entry [53]. Recently, a genome-wide CpG methylation profiling study showed MEN1-associated pNETs have a higher rate of hypermethylated CpG sites compared to VHL- or other pNET types, indicating menin contributes significantly to the epigenetic control of DNA methylation [54].

Overexpression of menin inhibits the growth of rat insulinoma cells, whereas its loss promotes their proliferation, supporting the tumor suppressor role of this gene [55]. These in vitro results were corroborated by studies showing development of pNETs in genetically engineered mouse models lacking *Men1* (Table 2). Menin can cooperate with other molecules to suppress pNET development. For instance, menin directly interacts with death-domain-associated protein (DAXX), another commonly mutated tumor suppressor gene in pNETs [23,24], to repress matrix metalloendopeptidase (MME), a zinc-dependent metalloprotease required for pNET cell proliferation, leading to pNET suppression [55]. Menin also cooperates with PTEN (Phosphatase and Tensin homolog), a tumor suppressive phosphatase that negatively regulates PI3K-Akt-mTOR signaling. The importance of menin-PTEN crosstalk in limiting pNET formation was demonstrated in conditional knockout mice lacking both *Men1* and *Pten* in pancreatic islet β cells. These mice had elevated PI3K-Akt-mTOR activity in tumors and developed pNETs at a much shorter latency than mice lacking either gene alone, suggesting menin-PTEN inhibition of the PI3K-Akt-mTOR pathway is crucial in preventing pNET pathogenesis [56].

### 2.2. PI3K-Akt-mTOR Pathway

Aberrant activation of oncogenic PI3K-Akt-mTOR pathway is implicated in both familial and sporadic pNETs. Inherited mutations in *TSC2* (Tuberous Sclerosis Complex 2) and *PTEN* tumor suppressors, key negative regulators of this oncogenic pathway, lead to autosomal dominant, multisystem disorders in which a small percentage of individuals develop pNETs [6,79]. The *TSC2* gene encodes a protein called tuberin that drives Rheb-GTP hydrolysis, thereby inhibiting mTORC1 activation [80]. Germline mutations in the *TSC2* gene leads to an autosomal dominant tumor predisposition syndrome called tuberous sclerosis (TSC), which is characterized by formation of benign tumors in almost every organ in the body [6]. In rare cases of TSC, patients develop functional (primarily gastrinomas and insulinomas) and non-functional pNETs [81,82]. LOH analysis of malignant pNETs reveal loss of the wild type *TSC2* copy with only the non-functional copy of the gene present at the disease locus [83]. A similar familial disease, called Cowden’s syndrome, is also associated with pNET development. Most patients with this syndrome inherit loss-of-function mutations in the *PTEN* gene, which results in Akt activation [84]. Although Cowden’s syndrome is characterized by tumors in the breast, thyroid, and endometrium, a minority of these patients develop pNETs driven by *PTEN* gene mutations [79,85].

The importance of PI3K-Akt-mTOR pathway activation in sporadic pNETs is well established and clinically relevant. Whole genome sequencing studies have revealed mutations in mTOR pathway genes including *PTEN*, *TSC2*, *PIK3CA,* and *DEPDC5* in 12–25% sporadic pNETs (Table 3) [36,37,38,39]. Moreover, allelic loss of chromosomal segments containing *TSC2* (16p) and *PTEN* (10q23) are observed in 25–36% of human pNETs [86,87]. Although mTOR pathway-related genetic mutations are less common, the percentage of sporadic pNETs with altered expression of the pathway members is remarkably high. mRNA expression profiling and IHC of *PTEN* and/or *TSC2* display downregulation of these genes in 75% sporadic pNETs, which correlates with disease progression and worse survival [88]. Aberrant cytoplasmic distribution of PTEN, which is primarily nuclear in normal islets, has also been reported in sporadic pNETs [87]. Mutations in mTOR pathway genes have been associated with poor prognosis in pNET patients [23], and hyperactivation of Akt and mTOR kinases has also been found in a subset of pNETs [89,90].

Findings from the tumor molecular profiling studies have been functionally validated using both in vitro and in vivo pNET models. Conditional loss of *Pten* in pancreatic islet cells leads to neuroendocrine tumor formation in mice (Table 2) [56]. Conversely, inhibitors targeting PI3K, Akt, or mTOR, either used alone or in combination, impede pNET cell growth and tumor development and metastasis in mice, suggesting pNET cells require Akt-mTOR signaling for their survival, growth, and migration [91,92,93,94]. Moreover, treatment of RIP-Tag2 mice (the first developed and prototypic transgenic pNET model) with an mTORC1 inhibitor, rapamycin, increases their average life span by 7 weeks (i.e., 16 weeks for males) [95]. Consistently, a rapamycin analogue—everolimus (RAD001)—was found to have potent anti-tumor activity in human pNET cell lines [96,97,98] and mouse models [12]. This provided a strong rationale for its use in clinical trials for advanced pNET patients [99,100]. In a phase III study (the RADIANT-3 trial) comprising 410 patients with advanced, low- or intermediate grade pNETs, everolimus treatment was associated with improved median PFS of patients compared to the placebo group (median PFS 11.0 vs. 4.6 months, *p* < 0.001), representing a 65% lower risk of disease progression or death in the everolimus treatment group [100]. A critical review of everolimus therapy for pNETs concluded it should be a first line therapy for patients with symptomatic, unresectable, insulin-secreting pNETs to control endocrine syndrome regardless of tumor growth [101].

Importantly, sustained inhibition of mTORC1 eliminates the ribosomal S6 kinase 1 (S6K1)/insulin receptor substrate-1 (IRS1) feedback loop, resulting in unwanted mTORC2-promoted Akt activation, which paradoxically enhances pNET growth and acquired resistance to mTORC1 inhibitors such as everolimus [102,103,104]. Therefore, the combination of an mTORC1 inhibitor with agents that prevent Akt activation, such as inhibitors of PI3K, Akt, mTORC2, or receptor tyrosine kinases, have been tested in various contexts and shown to have better outcomes [102,105].

### 2.3. INK4A/ARF Locus and RB1 Pathway

Mounting evidence implicates the role of the *INK4A*/*ARF* (originally called *CDKN2A*) gene locus and retinoblastoma 1 (RB1) tumor suppressor pathway in pNET pathogenesis. The *INK4A*/*ARF* locus encodes two different proteins, p16^INK4a^ and p14^ARF^ (mouse homologue p19^ARF^), derived from transcripts having distinct first exons but shared downstream exons that are translated in alternative reading frames [108,109]. As a result, the two protein products share no amino acid identity. The first product discovered, p16^INK4a^ (Inhibitor of CDK4/6), is a cyclin dependent kinase (CDK) inhibitor that enforces RB1 tumor suppressive activity by specifically inhibiting CDK4 and CDK6 [108]. By comparison, the other product, p14^ARF^ (Alternative Reading Frame protein), has numerous targets that it interacts with to prevent cancer [110]. ARF primarily activates the p53 tumor suppressor via MDM2 degradation, however, it also possesses many p53 independent tumor suppressive functions [110,111].

Inactivating p16^INK4a^ gene alterations are common in pNETs (Table 4). Several studies reveal homozygous deletions of the *INK4A* gene or hypermethylation of its promoter 5′ CpG island in the vast majority (up to 92%) of pNETs comprising gastrinomas and non-functional PNETs [103,104,112]. In general, insulinomas have a low frequency (17%) of INK4A genetic inactivation suggesting a pNET subtype-specific role of the gene [113]. Studies focusing on the mutational status of *INK4a*/*ARF* suggest mutations at this locus are absent in pNETs [24,112]. Nuclear staining of the p16^INK4a^ protein demonstrated loss of its expression in half of the analyzed pNETs [114], although interpreting loss of p16INK4a in tumors is always difficult since it is not expressed in most normal tissues due to tight transcriptional repression [115]. That said, silencing of *INK4A* in patient pNETs has clinical relevance as its hypermethylation occurs more commonly in malignant pNETs with metastases than in benign tumors [116]. Moreover, low p16^INK4a^ protein expression was associated with reduced survival in patients with G2-grade GEPNETs [117]. Mice lacking both p16^INK4a^ and p19^ARF^, or just p16^INK4a^ alone, spontaneously develop a spectrum of tumors but do not form NETs [118,119,120]. The likely reason is because the animals die of other, more aggressive tumors before NETs have time to develop. In neuroendocrine tissues, loss of p16^INK4a^ could be compensated by other CDK inhibitors that can enforce RB1 activity, such as p27^KIP1^ [113].

Unlike p16^INK4a^, promoter hypermethylation at the *ARF* gene is extremely rare in pNETs, although it is more commonly observed in other gastro-intestinal NETs [113]. Likewise, loss of *ARF* mRNA expression has been observed in a low percentage of non-functional pNETs [104]. However, the tumor suppressive role of p19^ARF^ has been demonstrated in a pNET mouse model of aggressive insulinomas. Ulanet et al., showed that loss of p19^ARF^ promotes pNET initiation and angiogenesis in RIP-Tag2 mice (Table 2) [121]. Since the tumors are driven by SV40 T antigen-mediated inactivation of RB1 and p53, the role of p19^ARF^ in this setting is independent of both tumor suppressors. Most recently, a tumor suppressive role for p19^ARF^ was implicated in a study of one of its interacting proteins, RABL6A, in pNET progression [77]. Genetic loss of *Rabl6* in RIP-Tag2 mice slowed pNET development of angiogenesis, which correlated with increased expression of p19^ARF^ protein in RABL6A-deficient tumors. RABL6A inactivation had no effect on *Arf* mRNA expression, suggesting part of RABL6A’s oncogenic effects may involve p19^ARF^ downregulation at the protein level.

Unlike advanced G3 pNECs (which have a high rate of *RB1* mutation), most well differentiated pNETs have an intact *RB1* gene [11]. Nevertheless, loss of RB1 tumor suppressor activity is critical for pNET development. Besides loss of p16^INK4a^ expression, RB1 activity can be impaired in pNETs via aberrant expression of upstream CDKs. Tang et al., showed that overexpression of CDK4 and its binding partner, cyclin D1, occurs in a majority of human pNETs, which correlated with elevated inactivating phosphorylation of RB1 [122]. Upregulated CDK expression and activity coincided with 19% of pNETs exhibiting amplification of both *CDK4* and *CDK6* genes. A more recent study of 267 patients demonstrated that high expression of cyclin D1, CDK4, and CDK6 proteins was associated with significantly increased Ki-67 in tumors [123].

Preclinical studies of CDK inhibitors in pNET cell lines and xenograft models have supported their use in treating pNET patients. Early studies with a non-selective kinase inhibitor targeting multiple CDKs (1/2/4/7), VEGFR and PDGFR, called ZK 304709, displayed significant anti-tumor activity (G2 phase cell cycle arrest and apoptosis) in BON1 xenograft tumors [124]. Others used a specific CDK4/6 inhibitor, palbociclib (PD0332991), and showed it induced a strong G1 phase arrest in cultured BON1 and QGP-1 cells as well as QGP-1 xenografts [122]. Another CDK4/6 inhibitor, ribociclib (LEE01), decreased BON1 and QGP-1 cell viability in a time and dose dependent manner [125]. That study found enhanced cell killing when ribociclib was combined with 5-fluorouracil or everolimus.

Clinical trials of CDK4/6 inhibitors in GEPNETs have yielded unsatisfying results. In a phase II (PALBONET) study, single agent palbociclib treatment failed to show beneficial outcomes in patients with metastatic grade 1 and 2 pNETs [127]. Patients in this study were not selected for molecular alterations and were heavily pretreated, with resistance linked to high levels of *CCNE1* and somatic *RB1* inactivating mutations. Establishing CDK4/6 and RB1 status as predictive biomarkers prior to treatment with these pathway inhibitors may be essential. Indeed, Keutgen et al., showed that non-functional pNETs associated with MEN1 and VHL syndromes have highly upregulated CDK4/6, which provides some rationale for selecting molecular targets for therapy based on pNET subtype [35]. In a pilot study by Dasari et al., (presented at ENETS conference, 2018), treatment of 18 patients having foregut NETs (55% of the cohort had pNETs) with ribociclib yielded no radiographic responses, no reduction in Ki-67 or phosphorylated RB1 in tumors, but did slightly improve the PFS of those patients. An ongoing, phase II trial of a more potent CDK4/6 inhibitor, abemaciclib, is currently being conducted in patients with advanced and refractory well-differentiated GEPNETs (NCT03891784). Most recently, combination of everolimus and ribociclib was found to have insufficient clinical activity to warrant further investigation in foregut well differentiated NETs [128].

While largely unremarkable, the clinical results with CDK4/6 inhibitors in pNET patients have highlighted several important points. Nearly all the trials were conducted as monotherapies. It is well established that in vivo inhibition of CDK4/6 alone using monotherapy approaches has cytostatic antitumor activity that can lead to acquired drug resistance through numerous mechanisms, including upregulation of CDK4, CDK6, cyclin E, CDK2 and/or activation of MEK [129]. This suggests that rational combination therapies simultaneously targeting known mediators of resistance may have greater success. In that regard, mechanistic studies providing deeper insight into factors controlling RB1 activity are warranted. Recent studies of a novel oncoprotein, RABL6A, showed it suppresses RB1 function in pNETs and other tumors by promoting CDK4/6 activity [129,130,131]. Mechanistic studies suggest RABL6A regulates the CDK4/6-RB1 pathway via multiple effectors, thereby providing novel options for combination therapies inhibitors that directly target CDK4/6 [132]. RABL6A was discovered as a binding partner of p14^ARF^ and subsequently shown to promote pNET cell survival and proliferation by inhibiting the RB1 pathway and activating Akt-mTOR oncogenic signaling [92,130]. Identification of new pNET targets, such as RABL6A and/or its effectors, may pave the way for developing combination therapies that will overcome poor pNET responses to CDK inhibitor monotherapy.

Genetically engineered mouse models have helped establish the functional role of the RB1 pathway in pNET pathogenesis. Loss of one *Rb1* allele caused moderate hyperplasia of the islet of Langerhans but was insufficient to drive islet cell tumorigenesis in mice [133]. However, when monoallelic loss of the *Rb1* gene is combined with mono- or bi-allelic loss of *Trp53*, pNETs are formed [134]. Those results suggest that loss of RB1 and p53 tumor suppressors cooperate to drive pNET pathogenesis. This theory is supported by the rapid development of islet cell tumors in RIP-Tag2 mice and Glu2-Tag mice (Table 2), both of which have silenced *p53* and *Rb1* by SV40 large T-antigen [57,70].

### 2.4. ATRX/DAXX

Mutations in *ATRX* (α-thalassemia/mental retardation syndrome X-linked) and *DAXX* (death domain associated protein) have been frequently observed in pNETs (Table 5) [11,36,37]. The ATRX protein is a component of heterochromatin bearing an ATPase/helicase-like domain characteristic of the SNF2 (sucrose non-fermentable 2) family of chromatin remodeling proteins [135,136]. Inactivating mutations in the *ATRX* gene lead to an X-linked condition called ATRX syndrome manifested as intellectual disability, α-thalassemia, genital abnormalities, and facial malformation [137,138]. Although ATRX is known to regulate gene expression by modifying chromatin, its exact function remains unclear [139].

DAXX is a pro-apoptotic protein that promotes c-Jun NH2-terminal kinase (JNK) pathway-induced apoptotic cell death by interacting with FAS and TGF-β (transforming growth factor-β) in the cytoplasm [136,140]. DAXX is primarily nuclear and associates with a tumor suppressor protein, promyelocytic leukemia (PML), at chromatin-bound PML-nuclear bodies (PML-NBs) that are involved in transcriptional regulation, cell apoptosis, and cell cycle [135,136]. In PML-NBs, DAXX has also been shown to interact with ATRX and inhibit its transcriptional repression activity [135,141]. The ATRX/DAXX complex orchestrates deposition of histone H3.3 and constitutes a novel chromatin remodeling complex at the pericentromeric and telomeric heterochromatin [139,142].

Whole exome sequencing of non-familial pNETs revealed inactivating-to-missense mutations in either *ATRX* or *DAXX* in up to 65% of tumors, predicting tumor suppressive roles in pNET pathogenesis [36,37,38,39]. The *ATRX* and *DAXX* mutations were mutually exclusive in those pNETs, in keeping with their functional involvement in the same pathway [24,40]. Immunohistochemical staining confirmed complete loss of ATRX or DAXX protein in the pNET samples harboring the corresponding gene mutations. Homozygous *ATRX*/*DAXX* mutations were observed only in a small fraction of the pNETs, however, the majority of tumors carrying heterozygous mutations are believed to lose the other non-mutant allele through gene deletion or epigenetic silencing [24].

Considering the role of ATRX/DAXX in modulating telomeric chromatin, Heaphy et al., performed telomere-specific fluorescence in situ hybridization (FISH) in 41 pNETs confirmed to have *ATRX*/*DAXX* mutations [143]. The majority (61%) of those pNETs displayed large, ultrabright telomere FISH signals, indicative of telomerase independent telomere modulation called alternative lengthening of telomeres (ALT). This positive correlation between *ATRX*/*DAXX* mutations and ALT in pNETs was similarly observed by others with one study suggesting these are late events in *MEN1*-associated pNET development [23,144].

Key pathways dysregulated by ATRX or DAXX loss in pNETs remain poorly understood. Feng et al., showed DAXX can directly interact with menin to epigenetically repress the expression of membrane metallo-endopeptidase (MME, also called CD10), a zinc dependent metalloprotease required for pNET cell proliferation [55]. Earlier studies in other cell types suggested DAXX negatively regulates p53, in apparent contradiction to its pro-apoptotic and tumor suppressive activities. Specifically, DAXX can inhibit p53 activity though direct interactions with p53 itself to block its transactivation of target genes or with a HAUSP-MDM2 complex to promotes MDM2 stabilization and downregulation of p53 expression [145,146]. Mouse modeling revealed no significant regulation of p53 signaling by DAXX in vivo but did solidify its role as a tumor suppressor since *Daxx*-deficient mice displayed more radiation-induced carcinomas than controls [147].

More recently, the same group developed mice with conditional Pdx1-Cre driven *Daxx* inactivation in the pancreas to better evaluate its function in pNETs (Table 2) [148]. *Daxx* loss alone had no effect on pancreas development and function, which remained normal, and surprisingly did not cooperate with *Men1* loss to enhance pNET pathogenesis. This suggested that, in mice, Daxx is not a strong endocrine tumor suppressor. On the other hand, loss of *Daxx* altered the transcriptome in association with derepression of endogenous retroviral elements (ERVs), which the authors found mirrors a similar dysregulation of genes near ERVs in patient pNETs with mutated *DAXX*. This creates a permissive chromatin state that cooperates with tissue stress, caused by inflammation or *Men1* loss for instance, to impair pancreas recovery following stress and potentially promote tumorigenesis. Future studies of ERV adjacent genes in human pNETs may provide meaningful insights into important mechanisms mediating effects of *DAXX* loss.

Human tumor studies have highlighted the pathological and prognostic significance of *ATRX*/*DAXX* mutations. pNETs having *ATRX*/*DAXX* or *MEN1* sporadic mutations were found to have an α-cell gene signature including high *ARX* (α cell-specific transcription factor) and low *PDX* (β cell-specific transcription factor) expression, which predicted worse recurrence-free survival of the patients [149]. Epigenomic and transcriptomic profiling was used to classify pNETs based on their *ARX* or *PDX* gene expression and found that *ARX*-positive pNETs with the ALT phenotype, characteristic of *ATRX*/*DAXX* mutation, have the shortest recurrence-free survival [150]. *ATRX*/*DAXX* mutation and the ALT phenotype also correlated with increased chromosomal instability, advanced tumor stage and metastasis, and reduced relapse-free survival [151]. In contrast to those studies associating *ATRX*/*DAXX* mutations with poor prognosis, Jiao et al., found that pNET patients having mutations in *ATRX*/*DAXX* (regardless of *MEN1* status) had better overall survival relative to others with the wild type genes [24]. Additional survival studies with larger and more diverse patient pools are warranted to clarify this discrepancy.

### 2.5. p53 Pathway

The *TP53* (*Trp53* in mice) gene, which encodes the p53 tumor suppressor, is rarely mutated in pNETs. However, evidence suggests loss of p53 activity via alterations in its regulators is critical for pNET genesis. p53 is a transcription factor involved in transactivation of genes that mediate DNA damage repair, cell growth arrest, cell senescence and apoptosis, among other antitumor processes. *TP53* gene alterations that limit p53 protein expression and activity contribute to the development and progression of a high proportion of human cancers [152,153]. Several key proteins are known to tightly regulate p53 expression and activity. Murine double minute 2 (MDM2) is an E3 ubiquitin ligase that catalyzes p53 ubiquitination leading to its proteasomal degradation, whereas its structural homologue, MDM4, inhibits p53 transcriptional activity via direct interaction [154]. Wild-type p53-induced phosphatase 1 (WIP1) is a serine threonine phosphatase that antagonizes p53. WIP1 dephosphorylates and stabilizes MDM2 and inhibits upstream activators of p53 such as ataxia telangiectasia mutated kinase (ATM), checkpoint kinase 1 (CHK1) and CHK2 [155,156].

Hu et al., showed that a high percentage of well-differentiated sporadic pNETs harbor amplifications of *MDM2* (22%), *MDM4* (30%), and *WIP1* (51%) genes, consistent with higher expression of their corresponding mRNAs and proteins (Table 6) [157]. Seventy percent of the pNETs in the study displayed amplifications of one or more of the above p53-inactivating genes. Moreover, these genetic alterations positively correlated with increased pNET progression to metastatic disease [157]. *PHLDA3* is a p53-transcriptional target and repressor of Akt activation found to have a tumor suppressive role in pNETs [158]. The same group observed LOH at the *PHLDA3* locus and aberrant promoter hypermethylation at the gene in over 70% pNETs, which correlated with poor prognosis in patients. Loss of *PHLDA3* overlapped with *MEN1* loss in a high proportion of pNETs in their study, suggesting they potentially regulate two different pathways that cooperate to suppress pNET development. Together, these studies demonstrate that p53 pathway inactivation, rather than *TP53* genetic mutation, is frequent in pNETs.

ATM is a tumor suppressor and activator of p53, whose low expression in pNETs has been associated with higher incidence of metastasis and poor prognosis [155]. Although *TP53* genetic mutation is reported in only 1–3% of pNETs [24,159], it is a regular event in poorly differentiated endocrine carcinomas of the gastrointestinal systems including pNECs [11]. Several groups report inactivating mutations in the *TP53* gene in over 90% of pNECs [11] or GEPNECs in general [160,161]. G3 pNETs and pNECs are both aggressive pNENs with an elevated Ki-67 index. In that regard, examining their *TP53* and *RB1* gene status could serve as an important distinguishing factor in their clinical diagnoses and prognosis [12].

The importance of reduced p53 tumor suppressive activity in driving pNET pathogenesis is supported by several genetically engineered pNET animal models (see Table 2). Combined inactivation of p53 and RB1 in pancreatic islet cells via SV40 large T antigen expression provided early evidence that their loss drives aggressive insulinoma development in mice [66,79,144]. In a β-cell polyoma middle T antigen (PyMT) transgenic model, *Trp53* deletion yielded primary pNETs in ~20% of mice while concurrent deletion of *Ink4a*/*Arf* doubled tumor incidence relative to loss of either tumor suppressor alone [73]. Since PyMT stimulates multiple oncogenic pathways including mitogen-activated protein kinase (MAPK), PI3K signaling and the Hippo pathway [162,163], and SV40 large T antigen inhibits both p53 and Rb, none of the above studies addressed the individual role of p53 loss in driving pNET formation. That was examined in a recent study, in which mice with pancreas-specific mutant p53 expression (Pdx1-Cre; Trp53^R172H^) failed to develop pNETs, although *Trp53* mutation greatly accelerated pNET progression to G3 pNETs when combined with *Rb1* deletion [75]. Likewise, Xu et al., found *Trp53* loss alone yielded no pNETs whereas its deletion with *Rb1* in the pancreas resulted in aggressive pNETs [76]. These findings show p53 inactivation alone is insufficient for pNET development; however, it cooperates with the loss of other tumor suppressors, particularly the p16^INK4A^/p19^ARF^-RB1 pathway, in promoting the disease.

### 2.6. VHL and Growth Factor Signaling

Germline mutations in the von Hippel-Lindau (*VHL*) tumor suppressor gene located on chromosome 3p25.5 cause an autosomal dominant tumor syndrome called VHL disease [164]. Patients with VHL develop diverse neoplasms that include hemangioblastoma of the central nervous system, retinal angiomas, renal cell carcinomas, and pheochromocytomas [165]. The common VHL-associated pancreatic lesions are cysts and cystadenomas, which are benign in nature and found in 35–75% individuals [165,166]. About 12–17% of VHL patients also develop pNETs, which are almost invariably non-functional and have metastatic potential correlating to the primary tumor size [167,168,169]. Lubensky et al., observed allelic deletion (LOH) of the wild type *VHL* gene in all the VHL pNETs in their study, bolstering the importance of this tumor suppressor in pNET pathogenesis [165,170].

VHL encodes a 232 amino acid protein, pVHL, which interacts with hypoxia inducible factor 1-alpha (HIF1-α) under normoxic conditions, targeting it for polyubiquitination and proteasomal degradation [171]. During hypoxia, HIF1-α translocates to the nucleus and conjugates with HIF-1β to form the heterodimeric HIF-1 transcription factor, which transactivates genes involved in cell proliferation, angiogenesis (e.g., vascular endothelial growth factor, *VEGF*), erythropoiesis, glucose homeostasis, and metastasis [172,173,174]. In VHL disease, the absence of pVHL causes constitutive stabilization and activation of HIF-1α irrespective of oxygen availability, leading to upregulation of HIF-1 target genes, essentially driving multi-organ tumorigenesis [175]. Of note, strong expression of HIF-1α and its transcriptional targets, carbonic anhydrase 9 and VEGF, was observed in early pNET stages suggesting these could be critical to pNET genesis in VHL patients [175]. Spiesky et al., reported that upregulation of several angiogenic genes, including VEGF and other HIF1-α targets, is required for cell cycle progression and cancer metastasis in VHL pNETs [176]. Exaggerated VEGF signaling caused by pVHL loss is consistent with the hyper-angiogenic phenotype of cancers arising in VHL individuals. Belzutifan (aka MK-6482), a HIF inhibitor, has recently been approved by FDA for use in adult VHL patients requiring treatment for associated pNETs, renal cell carcinoma, or central nervous system hemangioblastomas, however, not requiring immediate surgery (NCT03401788). Moreover, the efficacy and safety of Belzutifan monotherapy in patients with advanced pNETs or pheochromocytoma/paraganglioma are being studied in a new phase II clinical trial (NCT04924075).

Although the *VHL* gene per se is unaltered in sporadic pNETs, the genes and pathways downstream of pVHL or HIF1α play important roles in pNET pathogenesis. Immunostaining studies show positive expression of the VEGF (aka VEGF-A) protein in up to 85% of pNETs although intestinal NETs reportedly display higher VEGF expression per cell than pNETs (Table 7) [177,178,179]. Even when positive, the expression pattern of VEGF in pNETs is sparse, with only a small percentage of scattered tumor cells expressing the protein [178,180]. VEGF-C, another member of the VEGF protein family, is moderately to highly expressed in the majority of pNETs, with more than half of the cells in tumors staining for the protein [180]. Strikingly, VEGF-C expression was higher in pNET metastases than the primary tumors [180]. High expression of VEGF receptor-2 (VEGFR-2), the receptor for VEGF and VEGF-C, was observed in pNET endothelial cells suggesting VEGFR-2 mediates the angiogenic role of these specific VEGF proteins in pNETs [180]. Apart from growth signaling in endothelial cells, the VEGF family proteins can transduce autocrine signals required for proliferation, survival, and cell migration by binding to specific VEGFR sub-types expressed by the tumor cells [181]. In this regard, immunostaining studies have shown variable expression of VEGFR-2 and VEGFR-3 (which binds VEGF-C and -D) in pNET cells, implying autocrine signaling via these receptor tyrosine kinases could help promote pNET cell survival, proliferation, and metastasis [180].

Findings from pNET xenograft and genetic mouse models support the oncogenic role of VEGF in pNET development and progression. One group demonstrated that administration of bevacizumab, a VEGF monoclonal antibody, reduces endothelial cell proliferation and tubulogenesis in vitro and suppresses angiogenesis and growth of BON-1 cell derived xenografts in mice [177]. VEGF and its receptors are highly expressed throughout islet cell tumorigenesis in RIP-Tag2 mice, however, their levels are not elevated compared to normal islets [182]. Thus, it was suggested that VEGFR signaling was selectively increased in the hyper-proliferative, angiogenic islets, potentially via cross talk with other angiogenic HIF-1 targets [182]. Indeed, β-cell specific knockout of VEGF or blockade of VEGF receptor 2 (VEGFR2) disrupted initiation and progression of angiogenesis as well as tumor growth demonstrating the critical role of VEGF in pNET genesis [183,184]. Inhibition of VEGF signaling in RIP-Tag2 mice, however, is associated with rapid development of ‘adaptive resistance’ mediated by several mechanisms. One of these mechanisms is upregulation of other pro-angiogenic HIF-1 targets such as fibroblast growth factors (FGFs), as evidenced by sustained inhibition of tumor growth and angiogenesis by concomitant FGF and VEGF targeting [183].

The prognostic significance of VEGF expression levels and angiogenic status of pNETs seems to be context dependent. Zhang et al., showed that expression of VEGF correlates with micro-vessel density (MVD), a morphological gold standard of neo-vascularization, as well as increased metastasis and reduced PFS in GEPNETs [177]. This is consistent with other studies showing VEGF is a marker of progression and poor outcomes in GEPNECs [190]. However, a pNET-focused study by Couvelard et al., showed that although pNETs maintain high vasculature throughout their progression, they are associated with gradual decrease in VEGF expression and MVD as they progress [179]. In their immunohistochemical analysis, the low-grade benign pNETs unexpectedly displayed higher VEGF expression and MVD than the high-grade advanced pNETs. Moreover, low tumor MVD correlated with reduced survival in pNET patients, suggesting poor angiogenesis could be a marker of disease progression and poor prognosis, particularly in pNETs [179]. It is noteworthy that Zhang et al., analyzed low-grade, well-differentiated GEPNETs while Pavel et al., studied GEPNECs. By comparison, Couvelard et al., examined pNETs of varying histologic grade [185,187,191]. Therefore, observed discrepancies in the prognostic role of VEGF and angiogenesis could be greatly affected by differences in pNET types being analyzed.

Besides VEGF, other growth factors and their receptor tyrosine kinases have also been implicated in pNET development. Receptors of platelet derived growth factor (PDGF), namely PDGFR-α and -β, which transduce signals promoting angiogenesis and tumor cell proliferation, are elevated in VHL and sporadic pNET within both the tumor cells and the surrounding stroma [48,192,193]. In RIP-Tag2 mice, PDGFRs are exclusively expressed by the perivascular cells of the tumor vasculature and their pharmacological inhibition disrupts late-stage tumor growth [194]. However, PDGFR inhibition alone was less effective in preventing early stage angiogenic switch and tumorigenesis in RIP-Tag2 mice [194]. To that end, combined inhibition of PDGFRs and VEGFRs in RIP-Tag2 mice was more efficacious as it suppressed both angiogenic islet formation at early stages and tumor growth at later stages [194].

Consistent with the above findings, sunitinib (SU11248) maleate, a multitargeted receptor tyrosine kinase (RTK) inhibitor blocking VEGFRs, PDGFRs and stem-cell factor receptor (aka c-kit, expressed by more than 90% pNETs), displayed remarkable anti-tumor activity in RIP-Tag2 mice by disrupting angiogenesis and pericyte coverage of tumor vasculature [187,195]. The combination of sunitinib with a ‘chemo-switch’ regimen (i.e., initial phase of maximum tolerated dose followed by low maintenance dose) of cyclophosphamide induced remarkable tumor regression (97%) and improved median survival by >24 weeks in RIP-Tag2 mice [196]. Following successful phase I and II trials of sunitinib in pNET patients, a randomized, double-blind, placebo-controlled phase III trial was conducted in patients with advanced, well-differentiated pNETs [195]. Patients treated with a daily dose of 37.5 mg sunitinib had improved median PFS (11.4 vs. 5.5 months in the placebo group) and objective response rate (10% vs. 0% in the placebo group). Furthermore, a 10% death rate was observed in the sunitinib-treated patient group during the trial period compared to 25% in the placebo group.

Pazopanib is another multitargeted RTK inhibitor with activity against VEGFRs, PDGFRs, c-kit, and FGFRs. The clinical utility of this drug in patients with pNETs or metastatic GEPNETs has been tested in several phase II clinical trials, showing an overall response rate of 20% [197]. Surufatinib is a novel multitargeted RTK inhibitor that has recently been approved by FDA under ‘Fast Track Designation’ for the treatment of patients with advanced and progressive pNETs and non-pancreatic NETs (referred to as extra-pNETs/epNETs in the clinical trial) [192]. Surufatinib inhibits VEGFR1, VEGFR2, VEGFR3, FGFR1, and CSF1R (colony stimulating factor 1 receptor) and as such, disrupts tumor angiogenesis and promotes immune invasion. Two independent phase III clinical trials, SANET-p (NCT02589821) and SANET-ep, examined the effect of surufatinib in advanced pNET (n = 172) and extra-pNETs, respectively [193,198]. In these studies, surufatinib was found to significantly improve the median PFS of both pNET (10.9 vs. 3.7 months in the placebo group) and extra-pNET patients (9.2 vs. 3.8 months in the placebo group). Additional RTK inhibitors including Lenvatinib, axitinib, and sorafenib have also shown activity in phase II clinical trials [191,199].

In RIP-Tag2 mice treated with drugs targeting VEGF or VEFGR2, tumor cells rapidly develop ‘evasive resistance’ marked by increased invasiveness with peri-pancreatic lymph node and liver metastases [200]. This phenomenon is accompanied by upregulation of HIF-1α and its transcriptional targets due to vascular pruning, leading to intra-tumoral hypoxia plus upregulation and activation of hepatocyte growth factor (HGF) receptor, c-Met [201]. Combined inhibition of c-Met and VEGF using their selective inhibitors or a multi-targeted RTK inhibitor, such as cabozantinib, reduced pNET invasion and metastases. These data suggest exaggerated HGF-c-Met signaling drives malignant transformation of pNETs in RIP-Tag2 mice following VEGF-targeted treatments [201,202]. Activation of HGF-c-Met promotes tumor survival, proliferation, invasion, and metastasis in many cancers [202]. Krampitz et al., revealed c-Met is highly expressed in patient pNETs and is dependent on the paracrine action of its ligand, HGF, which is expressed only in the peripheral, normal tissues [203]. Because human c-Met cannot be activated by mouse HGF, growth of pNET patient derived xenografts (PDXs) in NSG mice required continuous administration of an exogenous c-Met agonist. This highlighted a critical role of c-Met stimulation in pNET cell engraftment and proliferation. The prognostic significance of c-Met expression was demonstrated by tissue microarray analysis showing that high expression of c-Met in pNETs correlates with poor patient survival [203].

Cabozantinib is a potent non-selective RTK inhibitor with activity against VEGFRs 1, 2, 3, and c-Met, along with other RTKs such as fms-like tyrosine 3 (FLT-3), RET, and AXL [197]. Because cabozantinib reduces pNET burden, invasion, and metastasis in RIP-Tag2 mice and promotes their survival [201], a two-cohort phase II clinical trial of this drug was carried out in patients with progressive, well-differentiated, grade 1–2 carcinoid NETs or pNETs [204]. In total, 3 of 20 pNET patients achieved partial response and the median survival of this cohort was 21.8 months. A randomized phase III clinical trial of cabozantinib in advanced or metastatic NETs is ongoing [197].

Exaggerated activity of epidermal growth factor receptor (EGFR) drives various cancers. pNETs and other GEPNETs generally lack activating mutations in the EGFR kinase domain [205]. Immunohistochemistry and qRT-PCR analyses revealed positive expression of EGFR or activated EGFR (p-EGFR) in 25–50% of primary pNETs or their metastases, which correlated with reduced survival [186,187,188]. EGFR inhibition by erlotinib alone attenuates aggressive pNET development and progression in RIP-Tag2 mice, eliciting a modest survival benefit [95]. When erlotinib is combined the mTOR inhibitor, rapamycin, the benefit is more profound. The adaptive resistance observed with rapamycin monotherapy is overcome by erlotinib and the survival of RIP-Tag2 mice is increased almost two-fold [95].

Downstream to RTK receptor signaling is the activation of multiple oncogenic pathways, including mTOR and MAPK signaling [94]. Insulin like growth factor-I (IGF-I) is another growth factor implicated in pNETs. von Wichert et al., revealed that BON-1 pNET cells release insulin-like growth factor-I (IGF-I) and express the IGF-I receptor [206]. Autocrine IGF-I signaling in these cells was found to promote chromogranin A release and mTOR and MAPK-dependent proliferation.

### 2.7. NF1 and RAS-RAF-MEK-ERK Pathway

Neurofibromatosis type I (NF1) is a genetic tumor predisposition syndrome with an incidence of 1 in 3000 individuals globally. All patients with NF1 almost invariably develop benign cutaneous neurofibromas [207]. Additionally, one-third of patients also develop enlarged benign plexiform neurofibromas, a subset of which progress into lethal nerve sarcomas called malignant peripheral nerve sheath tumors (MPNSTs) [207,208]. Less frequent tumor types in NF1 patients are juvenile myelomonocytic leukemia, pheochromocytoma (NET of the adrenal medulla), gastrointestinal stromal tumors, and rhabdomyosarcoma [207]. Up to 10% of NF1 individuals have also been found to develop pNETs, almost exclusively as periampullary duodenal somatostatinomas or in the form of pancreatic somatostatinomas, gastrinomas, insulinomas, or NF-pNETs in rare cases [6].

NF1 is an autosomal dominant disease caused by inheritance of one mutated copy of the *NF1* gene located on chromosome 17q11.2. *NF1* encodes a RAS GTPase-activating protein called neurofibromin [209,210,211], and tumorigenesis in NF1 individuals involves biallelic *NF1* inactivation [207]. Since neurofibromin negatively regulates RAS activity, its loss constitutively activates RAS GTPases, HRAS, NRAS, and KRAS, leading to aberrant activation of multiple downstream effector pathways including the oncogenic RAF-MEK-ERK (aka RAS-MAPK) signaling cascade.

Genetic alterations in signal transducers constituting or directly regulating the RAS-MAPK pathway are reportedly rare in pNETs. *KRAS*, one of the most commonly mutated genes in PDACs, was found to be unaltered in pNETs (Table 8) [24]. Likewise, related oncogenes HRAS, NRAS, and BRAF were mutated in less than 1% of pNETs [155]. Zakka et al., investigated specific gene mutations in circulating tumor DNA (ctDNA) present in liquid biopsies by next generation sequencing [212]. In their analysis of 165 pNET patients, *KRAS* mutations were seen in 63 (38%) patients and *NF1* mutations in 46 (28%) patients, which is remarkably higher than was previously reported from sequencing of primary tumor DNA. Comparative analysis of ctDNA vs. matched tissue DNA had exhibited variable concordance in the past [213,214]. Thus, Zakka et al., admitted the importance of validating their findings by studying the DNA of matched patient tumor tissues.

Regardless of mutational status, intact RAS-MAPK signaling is critical to pNET cell survival and growth. Treatment with inhibitors of RAF [215,216] or MEK [217,218] induced potent anti-tumoral effects in pNET cells. Moreover, combined inhibition of RAS-MAPK and PI3K-mTOR pathways synergized in reducing pNET cell viability and proliferation compared to targeting either pathway alone, suggesting cross talk between these pathways promotes pNET pathogenesis [219,220,221]. Interestingly, RAF activation with ZM336372 has also been shown to attenuate pNET cell growth [103,222,223]. In BON-1 pNET cells and other NET cell lines, ZM336372 promoted phosphorylation of RAF and its downstream effectors, MEK and ERK, but also caused GSK-3β inactivation and upregulation of the CDK inhibitors, p21 and p18 [224]. As such, ZM336372 treatment resulted in reduced cell proliferation and downregulation of NET markers, CgA, and achaete-scute complex-like 1 (ASCL1). Based on these findings, the anti-tumoral effects of RAF activation are believed to be independent of the RAF-MAPK pathway [225].

### 2.8. Somatostatin Receptor Signaling

Somatostatin receptor (SSTR) signaling has anti-secretory and anti-proliferative roles in NETs. Somatostatin (SST) is an endogenous cyclic peptide hormone concentrated in the central nervous system, pancreas, and GI tract [226,227]. In the pancreas, SST is secreted by the islet δ cells and inhibits the release of insulin (from β cells), glucagon (from α cells), and pancreatic amylase (from exocrine cells). In the GI tract, SST inhibits the release of several enzymes and hormones including serotonin, gastric acid, gastrin, cholecystokinin, vasoactive intestinal peptide, and secretin. Moreover, SST has demonstrated anti-proliferative functions in tumors arising from the pancreas and the GI tract [219,226,227,228]. This antitumor activity is key to the development of several NET therapies.

There are two active biological forms of SST, one with 14 amino acids (SST-14) and the other 28 (SST-28), both produced by the enzymatic cleavage of the precursor protein prosomatostatin [226]. SST-14 is more commonly produced whereas SST-28 is more potent, although their functions greatly overlap. Five different SST receptors, SSTR1-5, bind these SSTs. SST-14 binds with higher affinity to SSTRs 1-4 while SST-28 is more selective for SSTR5 [220]. SSTRs are coupled to the Gi-protein, as such their anti-secretory action is primarily mediated by the inhibition of adenylate cyclase leading to decreased cAMP production [220,226]. SSTR2 is widely considered the most important mediator of SST antiproliferative functions, which include induction of cell cycle arrest and apoptosis as well as inhibition of tumor angiogenesis and growth factor (e.g., VEGF and IGF-1) expression [226]. Mechanistically, SSTRs bind and activate several protein tyrosine phosphatases (PTPs), small heterodimer partner 1 (SHP1), SHP2 and PTPη, via cytosolic src homology 2 (SH2) domains in the PTPs. Activation of PTPs causes dephosphorylation of tyrosine kinase receptors and their substrates and subsequent inhibition of important oncogenic pathways, such as Ras-MAPK and PI3K-Akt-mTOR signaling (Figure 1). Furthermore, SSTR2 activation can cause tumor cell arrest by upregulating the cyclin dependent kinase inhibitor, p27, by a mechanism involving SHP-1 activation [221,222]. SSTR2 also induces tumor cell apoptosis by several mechanisms including upregulation of death receptor 4 and tumor necrosis receptor 1, and downregulation of the anti-apoptotic protein, Bcl-2 [223].

SSTRs, mainly 2 and 5, are abundantly expressed in up to 90% of GEPNETs (Table 9) [97,196,229,230], including increased SSTR2 and SSTR5 levels in the vast majority of pNETs [88,185]. This SSTR upregulation in NET cells facilitates SSTR targeting by SST analogues (SSAs) to limit tumor cell hormone secretion and proliferation while additionally enabling use of radiolabeled SSAs for NET imaging and therapy [15].

In vitro mechanistic studies have associated SST-SSTR signaling with other established pNET pathways. For instance, tumor suppressive TGF-β signaling induces SST expression in BON-1 cells, which promotes the SST-SSTR2 anti-proliferative autocrine loop [231]. Intriguingly, inhibition of oncogenic HDACs in pNET cells that express low endogenous SSTR2 levels (e.g., BON-1) functionally upregulates SSTR2 at the cell surface through increased transcription and translation of the receptor, potentially via Notch1 activation [232,233,234]. These clinically relevant findings may help establish new strategies to improve SSTR2-based imaging and therapy in pNETs. Notably, SSTR2 expression has prognostic significance in GEPNETs. Nodal and hepatic metastases were found to exhibit significantly lower SSTR2 expression compared to the primary pNETs [235]. Conversely, higher SSTR2 expression is a predictor of better patient overall survival and correlates with longer PFS following SSA therapy [229,236,237,238].

Since somatostatin has a very short half-life of 3 min [242], synthetic SSAs (e.g., octreotide and lanreotide) have been developed for clinical use in the first line treatment of unresectable, metastatic pNETs [243,244]. SSAs are effective in controlling the hormonal secretion and growth of functional pNETs and small bowel (SB)/carcinoid NETs, thereby alleviating the clinical symptoms precipitated by tumor-related hormone hypersecretion. In the PROMID (placebo-controlled, prospective, randomized study in patients with metastatic neuroendocrine midgut tumors) trial, octreotide significantly prolonged the PFS from 6.0 months in the placebo group to 14.3 months, validating the clinical benefit of SSTR targeting [243,244]. The subsequent CLARINET (controlled study of lanreotide antiproliferative response in GEPNETs including those in the pancreas) trial showed that lanreotide significantly prolongs the PFS in patients with advanced, G1/G2 differentiated, non-functioning, SSTR-positive NETs (PFS at 24 months 65.1% in the lanreotide group vs. 33% in the placebo group) [245]. Furthermore, the combination of everolimus and octreotide has been shown to improve the median PFS (from 9.7 to 16.7 months) and clinical benefit rates compared to monotherapy with everolimus or octreotide alone [110,246,247].

A new broader spectrum SSA, pasireotide, binds SSTR1, 2, 3, and 5 [227]. Pasireotide possesses more potent anti-proliferative activity compared to octreotide in cultured cells and in mice [248,249,250]. When used as a first line therapy in patients with advanced NETs, pasireotide displayed long-lasting anti-tumor control efficacy (PFS 11 months) [251] and provided an improved tumor control rate at 6 months compared to octreotide (though not statistically significant) [252]. The use of pasireotide is, however, limited by higher incidence of hyperglycemia in patients compared to octreotide. The COOPERATE trial showed that the combination of pasireotide with everolimus in patients with progressive G1 through G2 pNETs provides higher response rates compared to everolimus alone; however, no significant differences in overall survival and PFS were observed between the two experimental cohorts [253].

Beyond cold (non-radiolabeled) SSAs, peptide receptor radionuclide therapy (PRRT) using radioisotope-labeled (“hot”) SSAs (e.g., DOTA peptides-DOTATATE and DOTATOC) has emerged as a highly effective strategy to image and treat metastatic, well-differentiated G1 and G2 GEPNETs [227]. Administration of ^111^In-pentetreotide or ^68^Ga-DOTATATE/DOTATOC has facilitated the highly sensitive single photon emission computed tomography (SPECT) or positron emission tomography (PET) imaging of GEPNETs [246,254,255]. The first prospective randomized study treating patients with progressive metastatic midgut NETs by PRRT, NETTER-1, reported better PFS (20-month PFS at 65.2% vs. 10.8%) and improved response rate (18% vs. 3%) with ^177^Lu-DOTATATE (administered with or without octreotide LAR) compared to high-dose octreotide alone [247]. However, pNET patients were not included in this study. Several other studies have observed significant tumor control rates in pNET patients treated with PRRT [15]. Zandee et al., reported that ^177^Lu-DOTATATE treatment in 34 patients with metastatic, functional G1-G2 pNET patients resulted in partial or complete response in 59% of patients, disease control in 78% and reduction of symptoms in more than 80% [17]. A retrospective study assessing the efficacy of PRRT (using ^68^Ga-, ^111^In-, or ^99m^Tc-based SSAs) in 149 GEPNEN G3 patients (including 89 with primary pNETs) demonstrated promising response rates (complete or partial response in ~42% patients), PFS (14 months), and overall survival (29 months) in patients, with comparatively better outcomes noticed in those having progressive disease [256]. Considering PRRT can cause adverse effects including nephrotoxicity and bone marrow suppression, such therapy requires careful monitoring of patients during and after treatment.

### 2.9. Miscellaneous Genes and Pathways

#### 2.9.1. Myc

Myc (or cellular Myc, c-Myc) is a potent oncogene known to drive various cancers. Functionally, Myc is a transcription factor that regulates the expression of numerous genes involved in cell proliferation, cell cycle progression, differentiation, angiogenesis, metabolism and apoptosis [257,258,259]. Although the *MYC* gene is rarely altered in patient pNETs, several studies highlight the role of Myc protein in pNET development. Patient tumor analyses show that almost all pNETs (80–100%) exhibit strong immunoreactivity for Myc (Table 10) [107,260].

Expression or activation of transgenic *Myc* can induce pNET development in animal models [83,261,262]. Pelengaris et al., designed a reversibly switchable pNET mouse model (plns-c-MycER^TAM^) in which transgenic Myc is expressed under an insulin promoter (*plns*) and its activation can be induced by tamoxifen (TAM) administration (Table 2). Activation of Myc leads to hyperproliferation of pancreatic β cells in these mice, although it is rapidly counterbalanced by Myc-induced apoptosis [74]. Co-expression of an antiapoptotic Bcl-2 family protein-Bcl-x_L_, however, causes mice to progressively develop angiogenic, invasive islet tumors [74]. Dependence on Myc for the tumor phenotype was shown by the fact that tumors undergo regression as a result of vascular degeneration and β-cell apoptosis when Myc is deactivated. Others showed that targeted expression of Myc in pancreatic progenitor and islet cells achieved through somatic delivery of the oncogene at post-natal day 2 (P2) induces pNET development by 7 months in p16^INK4a^/p14^ARF^ null mice, but not in mice which are wild-type for both p16^INK4a^/p14^ARF^ [263]. In zebrafish, targeted expression of *MYCN* in pancreatic β-cells induces pNECs that resemble the human disease, suggesting other members of the Myc oncogene family may be involved in pNET pathogenesis [264].

Myc cross-talks with several key pNET pathways, in part because it is an important downstream target of mTOR in many cancers, including pNETs [107,265]. In that regard, inhibition of Myc through shRNA or pharmacologic (10058F4, CPI-203) approaches enhances the sensitivity of pNETs to mTOR inhibitors and reverses pNET resistance to mTOR inhibition by suppressing Akt activation [107,266]. Myc also drives tumor angiogenesis by upregulating VEGF (one of its transcriptional targets) and other angiogenic proteins [267,268]. Consistent with those observations, Myc inhibition disrupts the pNET vasculature and causes tumor regression in Myc-driven pNET mouse models [74] and in RIP-Tag2 mice [269].

#### 2.9.2. Src Family Kinases

Src family kinases (SFKs) promote DNA synthesis, cell cycle progression, angiogenesis, and cell motility by activating Myc, Ras, VEGF, and mTOR driven pathways [277].

Although the oncogenic role of SFKs has been well established in several solid cancers including breast, prostate, colon, and pancreatic cancers, studies demonstrating their relevance in pNET pathogenesis are limited [277]. Early studies showed that lymphocyte specific protein tyrosine kinase (LCK), which is a SFK, is highly upregulated at both the mRNA and protein level in primary pNETs, pNET metastases, and cell lines, BON-1 and QGP-1 (Table 10) [270]. c-Src, which is the prototype and most-studied SFK, activates the mTOR pathway in pNETs [278]. Conversely, loss of endogenous Src activity using an inhibitor (PP2) or RNAi decreases the phosphorylation of mTOR downstream targets, rpS6 and 4-EBP1, in BON-1 and QGP-1 pNET cells [278]. Concomitant inhibition of c-Src (by PP2) and mTOR (by everolimus) significantly impairs pNET cell growth compared to targeting either protein alone. Of note, this combination therapy also blocks feedback activation of the PI3K-Akt loop responsible for pNET resistance to mTOR monotherapy [278], highlighting the potential benefit of co-targeting Src and mTOR signaling in these tumors. More studies investigating the in vivo efficacy of SFK inhibitors alone or in combination with mTOR inhibitors in pNET models could have high clinical relevance but warrant deeper analyses of SFK status in patient pNETs [277].

#### 2.9.3. RABL6A

RABL6A is a novel oncogene first discovered as a binding partner of the p14^ARF^ tumor suppressor [279]. It is a Rab-like GTPase that has several different synonyms in the literature, including C9orf86, PARF, and RBEL1 [279,280,281]. Expression of RABL6A correlates with poor survival in breast cancer [282], pancreatic ductal adenocarcinoma [283] and non-small cell lung cancer [284,285]. RABL6A is highly expressed at the protein and genetic level in patient pNETs (Table 10) [106,130]. Functional studies demonstrated that RABL6A promotes pNET cell survival and proliferation by regulating important pNET pathways including inhibition of RB1 [130] as well as activation of Akt-mTOR via inhibition of protein phosphatase 2A (PP2A) [92]. Notably, pathway analysis of microarray results in BON-1 cells suggests RABL6A upregulates Myc and VEGFR signaling genes in pNETs [130]. Most recently, the role of RABL6A as a pNET driver has been corroborated by in vivo studies revealing that RABL6A promotes tumor growth and the angiogenic switch in a transgenic (RIP-Tag2) pNET mouse model [77].

#### 2.9.4. HDACs

Histone deacetylases (HDACs) promote chromatin compaction and inaccessibility of the promoter regions leading to gene silencing [271]. Epigenetic silencing of numerous tumor suppressor genes due to exaggerated activity of HDACs have been implicated in the carcinogenesis of multiple organs, including the exocrine and endocrine pancreas [286]. A comprehensive immunohistochemical analysis of HDACs in 57 pNETs resected between 1997 and 2013 revealed significant upregulation of all 5 HDAC classes (I, IIA, IIB, III, and IV) in pNETs compared to the corresponding pancreatic tissues (Table 10) [271]. The study also found a correlation between specific HDAC expression with pNET grade and patient survival, highlighting a predictive role HDACs can play in determining pNET therapy and outcome [271]. The same group also showed that the expression of HDAC-3 (class I) and -4 (class IIa) significantly correlates with that of miRNA449a, which was found to be a maker of proliferation status and patient survival in pNETs [287].

Alvarez et al., employed RNA-seq followed by MARINa (Master Regulator Inference algorithm) and VIPER (Virtual Proteomics by Enriched Regulon analysis) algorithms on a cohort of 212 patient GEP-NETs to identify master regulator proteins driving neuroendocrine lineage progenitor state and immune-evasion in these tumors [261]. Their model predicted the class I HDAC inhibitor, entinostat, to be a potent inhibitor of master regulator activity in 42% of metastatic pNETs. This led the authors to validate the growth suppressive activity of entinostat in a mouse xenograft model of pNETs although its anti-metastatic activity was not assessed.

Scott et al., performed RNA-seq on primary pNETs and their matched liver and lymph node metastases to identify metastatic gene signatures. Ingenuity pathway analysis (IPA) and Connectivity Map (cMAP) of 902 differentially expressed genes identified HDAC as one of the top metastasis-specific pNET drug targets [106]. In vitro testing of class I HDAC inhibitors, entinostat and mocetinostat, on BON-1 and QGP-1 cell lines showed moderate anti-proliferative activity [106]. Functional inhibition of class IIA HDACs, HDAC 4 and 5 by LMK-235 has also been shown to reduce the viability and promote apoptosis of BON-1 and QGP-1 cells [233]. At this point, assays measuring the anti-metastatic activities of HDAC inhibitors in pre-clinical pNET models are needed. It will be important to compare and contrast different HDAC inhibitors since some can promote metastasis of other tumor cell types via PKC activation and HDAC11 inhibition [288,289], underscoring the importance of thorough drug testing and validation.

Valproic acid (VPA) is another example of an HDAC inhibitor that can reduce BON-1 and H727 (human pulmonary carcinoid) NET growth, biomarker expression, and hormone secretion [290]. Its activity was linked to activated Notch1 signaling. VPA also disrupted the growth of mouse tumor xenografts derived from these carcinoid cell lines [290]. A pilot phase II study of VPA in patients with low-grade carcinoid and pancreatic NETs demonstrated stable disease as the best response in only half patients [291]. A phase II trial (NCT00985946) of panobinostat, a pan-HDAC inhibitor, in 15 patients with low-grade NETs also resulted in a low response rate although high rate of stable disease and median PFS of 9.9 months were achieved [292]. Entinostat is currently being tested in phase II trials in patients with relapsed or refractory abdominal NETs (NCT03211988). As described in a prior section on somatostatin receptor (SSTR) signaling, combination therapies targeting both HDACs (where inhibitors upregulate SSTR levels) and SSTRs together may have greater efficacy than either therapy alone.

#### 2.9.5. Heat Shock Protein (HSP) 90

Heat shock protein 90 is a molecular chaperone required to maintain stability and activity of diverse proteins regulating cell signaling, proliferation, survival, and carcinogenesis [293]. HSP90 is highly expressed in non-pancreatic carcinoid NETs [294], and in 75% of primary pNETs and their metastases (Table 10) [185]. Several HSP90 inhibitors exhibit growth inhibitory effects in pNET cell lines [185,294,295,296]. HSP90 inhibitors, 17-AAG [185], IPI-504 [296], AUY922, and HSP990 [295] not only induced apoptosis and inhibited proliferation of human pNET cell lines in vitro and in vivo, but also downregulated the expression of important pNET growth factors such as EGFR, IGF1R, and VEGFR2. This correlated with reduced activity of downstream ERK and Akt-mTOR oncogenic pathways. Furthermore, combined inhibition of HSP90 and Akt or mTOR produced an additive anti-neoplastic effect in pNET cells and overcame IGF1R-dependent feedback activation of Akt associated with sustained mTOR inhibition [296].

#### 2.9.6. Aurora Kinase

Aurora kinases A, B, and C are serine-threonine kinases that regulate chromosomal assembly and segregation during mitosis [297]. Overexpression of aurora kinases A and B has been reported in a variety of solid cancers, including prostate, colon, pancreas (exocrine), breast and thyroid cancers [155]. Of all members in this kinase family, aurora kinase A has the most well-characterized oncogenic role. It is characterized by downregulation of the p53 tumor suppressor via MDM2 activation [298], inhibition of GSK3-β, and activation of β-catenin and SRC [299]. In an IHC analysis of patient NETs, aurora kinase A was expressed in 8 of 10 insulinomas, 100% of 13 nonfunctional pNETs, and 20 SBNETs while being absent in the surrounding non-cancerous tissue and control normal pancreas (Table 10) [262]. That study and others have shown that both BON-1 and QGP-1 pNET cell lines express aurora kinase A and B [262,300], and treatment with a pan-aurora kinase inhibitor, danusertib, inhibits pNET cell proliferation at nanomolar concentrations. Danusertib also disrupted primary tumor growth in a murine xenograft model and halted development of pNET cell derived liver metastases following intrasplenic injections [262]. In agreement, Hofving et al., showed that aurora kinase inhibitors strongly attenuate pNET cell viability [301]. Briest et al., (2018) found that nuclear immunoreactivity against aurora kinase B in GEPNETs correlates positively with tumor grade and size, albeit negatively with their differentiation and functionality [302]. A selective aurora B inhibitor, ZM447439, potently suppresses proliferation and induces apoptosis of BON-1 and QGP-1 pNET cells and exhibits potentiated activity when combined with cisplatin or streptozotocin [300].

Although pNET expression of aurora kinases and their requirement for pNET cell growth in vitro and in vivo have been demonstrated in multiple studies, the exact mechanisms by which these kinases promote pNET pathogenesis and their interplay with other canonical pNET pathways is yet to be fully understood.

#### 2.9.7. Developmental Pathways

(i) Hedgehog signaling

Hedgehog (Hh) signaling is a highly conserved developmental pathway in vertebrates with important roles in cell cycle regulation and tumorigenesis [303]. In Hh signaling, binding of Desert, Indian, and Sonic Hh ligands with Patched (Ptch-1), a transmembrane receptor, relieves the inhibition of Smoothened (Smo) by Ptch-1 [304]. Activated Smo cooperates with Gli transcription factors, Gli1 and 2, to transactivate pro-proliferative genes including Cyclin D, Cyclin E, Myc, and Gli1 [303,304]. Hh signaling also induces expression of transcription factors such as Snail, Snug and Twist, which mediate epithelial mesenchymal transition (EMT) of metastatic tumor cells [303].

Several reports have highlighted the role of Hh signaling in NETs and NECs. Studies based on qRT-PCR and immunohistochemical analyses showed that Gli1, Ptch1, Sonic Hh (Shh), and Snail are highly expressed in gastrointestinal (GI) NECs [305] and SBNETs [306] while modestly expressed or absent in GI carcinoids and adenocarcinomas [305]. Disruption of Hh signaling by a Smo inhibitor, cyclopamine, suppresses NEC cell survival, proliferation, and invasion in a manner that correlates with downregulation of Gli1, Ptch1, hAsh (a bHLH transcription factor regulating neuroendocrine differentiation), and Snail1 as well as upregulation of E-cadherin [305,307].

Hh signaling is critical to normal embryonic development and functioning of the endocrine pancreas [308]. Nevertheless, limited studies have investigated the expression or activation status of Hh pathway in patient pNETs. Fendrich et al., revealed that pancreatic gastrinomas and their lymph node metastases, unlike primary and metastatic duodenal gastrinomas, lack detectable expression of Shh, undermining the potential importance of Shh signaling in this pNET subtype [309]. Gurung et al., found expression of Ptch1 in over 50% of sporadic or MEN-1 associated patient pNETs without clear prognostic significance (Table 10) [272]. Gli transcription factors (1, 2, and 3) and Ptch are expressed in BON-1 cells, which exhibit reduced growth upon cyclopamine treatment, suggesting Hh signaling promotes pNET cell proliferation in vitro [306]. In RIP-Tag2 pNET mice, administration of cyclopamine by intraperitoneal injection [310] or of a new, orally bioavailable Smo antagonist LDE225 [311] induces pNET cell apoptosis and reduces cell proliferation and tumor burden, improving their survival by 1–3 weeks.

Evidence suggests Hh signaling is negatively regulated by menin and that this molecular crosstalk could be exploited to treat MEN-1 tumor syndrome [312]. Menin recruits protein arginine methyltransferase 5 (PRMT5) to the *Gas1* gene promoter and downregulates Gas1 via PRMT5-catalyzed repressive histone arginine symmetric demethylation [313]. Gas1 is critical to the binding of Shh ligand with its receptor Ptch1. Thereby, loss of menin in MEN-1 disease promotes Gas1 upregulation and subsequent Hh pathway activation [313]. Consistent with those findings, increased expression of Gas1, Gli1, and Ptch1, which is indicative of activated Hh signaling, was observed in primary islets from *Men1^l^*^/*l*^; *RIP-Cre* mice compared to the WT controls [313]. Furthermore, inhibition of Hh signaling by a Smo antagonist, GC-0449 (aka vismodegib), decreases proliferation and insulin secretion of insulinomas developed by *Men1^l^*^/*l*^; *RIP-Cre* mice [312,313]. These studies suggest that agents targeting Hh signaling, some of which are FDA approved for other cancers (vismodegib for basal cell carcinoma), could hold a significant promise in MEN-1 therapy.

(ii) Notch1 signaling

Notch signaling comprises four transmembrane Notch receptors (Notch 1–4) activated by five Notch ligands (Delta-like ligand 1 [DLL1], DLL3 and DLL4, and Jagged1 and Jagged2) [314,315]. Notch signaling has an evolutionarily conserved role in cell-fate-determination and is known to regulate the differentiation of stem cells and progenitor cells in the development of pancreas [314].

Notch signaling in cancer can have either oncogenic or tumor suppressive outcomes depending on the tissue type [155]. Activation of Notch is required for cancer stem cell maintenance in ovarian cancer, colon cancer, lung adenocarcinoma, breast cancer, and pancreatic cancer [314]. However, in GEPNETs the Notch pathway putatively acts as a tumor suppressor based on the evidence that Notch activity or expression is greatly reduced whereas its reactivation induces the anti-tumoral effects [316,317]. Notch suppresses neuroendocrine differentiation of the developing endoderm by inhibiting the expression of bHLH transcription factors (e.g., Ascl1), known to drive the process [318,319]. Nakakura et al., (2005) showed that Ascl1 is upregulated in GI NETs and BON-1 cells. Exogenous overexpression [318,320] or pharmacological activation [223,299,321,322] of Notch1 induces loss of Ascl1, reduction in neuroendocrine markers (synaptophysin and chromogranin), decreased serotonin production by repression of tryptophan hydroxylase (TPH1), and growth inhibition of BON-1 and H727 cells. The HDAC inhibitor, VPA, can also upregulate Notch1 in NET cells through unclear mechanisms [323]. In a phase II pilot trial conducted in eight patients with low grade NETs (six carcinoid and two pancreatic), VPA increased Notch1 mRNA (which was absent prior to treatment), decreased chromogranin A levels and induced stable disease as best response in four patients [291].

(iii) Wnt/β-catenin signaling

The canonical Wnt signaling cascade is a tumorigenic pathway that involves: (1) binding of Wnt ligands with a receptor complex consisting of the primary receptor Frizzled (Fz) and a co-receptor lipoprotein receptor-related protein 5/6 (LRP5/6); (2) subsequent activation of a cytoplasmic protein called dishevelled homologue (Dv1); and (3) inhibition of the β-catenin destruction complex comprising axin, adenomatous polyposis coli (APC) and glycogen synthase kinase 3β (GSK3β) by activated Dv1, leading to β-catenin stabilization, its nuclear localization, and formation of β-catenin/T-cell-specific transcription factor (TCF)/lymphoid-enhancer binding factor (LEF) transcriptional complex [315]. Target genes of the β-catenin/TCF/LEF complex (e.g., Myc, Cyclin D, gastrin, VEGF, Met) are involved in promoting carcinogenesis, tumor invasion, and metastasis.

Wnt/β-catenin signaling regulates normal development of the pancreas [324]. Several groups have thereby investigated its role in pNET genesis. Weiss et al., observed strong membranous β-catenin immunostaining in 100% of 13 stage III/IV pNETs (as per TNM staging) and in only 47% (35/74) of stage I/II tumors (Table 10) [273]. Moreover, 15% of stage III/IV pNETs and none of stage I/II tumors immunostained for nuclear β-catenin. Although the β-catenin expression pattern was influenced by tumor staging, no correlation was found with tumor grade and disease-specific survival (DSS). The authors also found loss of APC, a member of β-catenin degradation complex, in 70% of total pNETs without any association with tumor stage, grade, or DSS [273]. Downregulation of several other Wnt/β-catenin pathway inhibitors has been reported. Kim et al., demonstrated that reduced expression of *SFRP-1*, *Axin-2*, *DKK-1*, *DKK-3*, and *WIF-1* in BON-1 cells is mediated by CpG hypermethylation or H2K9 dimethylation of the corresponding genes, highlighting the importance of epigenetic regulation in pNET Wnt signaling [325].

Pharmacological inhibition of Wnt/β-catenin signaling or overexpression of its endogenous inhibitors reduces viability, proliferation, and tumorigenicity of pNET cells in vitro and in vivo [273,326]. Some novel Wnt/β-catenin signaling activators or downstream transcriptional targets have been implicated in pNET pathogenesis. Tenascin-C (TNC), an extracellular matrix molecule protein, activates Wnt/β-catenin signaling via downregulation of DKK1, a Wnt pathway inhibitor, thereby promoting pNET cell survival, angiogenic switch, tumor progression, and lung micrometastasis in RIP-Tag2 mice [327]. Consistent with those data, TNC expression in human insulinomas was found to correlate with metastasis [327]. Kim et al., showed Neurotensin is a direct Wnt/β-catenin signaling transcriptional target that promotes pNET cell proliferation, anchorage-dependent growth, and the expression of growth-related proteins, cyclin-D1 and Myc [328].

Wnt/β-catenin signaling is regulated by menin in pNETs. Jiang et al., reported that menin promotes β-catenin phosphorylation and degradation in mouse and human pNETs [329]. They also showed that genetic ablation or pharmacological inhibition of β-catenin attenuates pNET growth and the hypoglycemic phenotype in β-cell specific *Men-1* knockout mice. This coincided with downregulation of pro-proliferative β-catenin target genes, *Ccnd1*, *Myc*, and *Mcm2* in the tumors and improved mouse survival. These results suggested that suppression of oncogenic β-catenin signaling partly accounts for the tumor suppressive role of menin in pNETs. In contrast, Chen et al., showed that menin directly interacts with β-catenin, promotes β-catenin transcriptional activity in TGP61 mouse islet tumor cells, and selectively upregulates *Axin2* (but no other Wnt/β-catenin signaling targets tested), consistent with their observation of increased H3K4 trimethylation at the *Axin2* gene promoter following menin overexpression [330]. Taken together, these results suggest that menin regulation of Wnt/β-catenin signaling is complex. It is likely context-dependent and may affect the expression of Wnt target genes in a biased fashion [330].

(iv) TGF-β and SMADs

Transforming growth factor-β (TGF-β) superfamily proteins, TGF-β_1_, β_2_, and β_3_, interact with transmembrane receptor serine threonine kinases, TGF-βRI and TGF-βRII, to phosphorylate and activate key downstream transcription factors, SMAD2-4 [321,331]. Target genes of SMADs regulate cell differentiation (including that of the endocrine pancreas) and proliferation [322,331]. Activation of TGF-β signaling can induce or inhibit cell proliferation depending on the state of cell differentiation or transformation [331]. In pNETs, however, TGF-β_1_ signaling has been shown to inhibit cell growth through upregulation of the p21^WAF1/CIP1^ tumor suppressor, which is a SMAD-target gene [274,331].

Immunohistochemical studies revealed positive expression of TGF-β_1_ and its receptors, TGF-βRI and TGF-βRII, in most (75–100%) patient pNETs as well as in BON-1 and QGP-1 pNET cell lines (Table 10) [185,274]. TGF-β_1_ treatment inhibits BON-1 cell growth by promoting cell cycle arrest, concomitant with p21^WAF1/CIP1^ induction and Myc downregulation [274]. Conversely, treatment with anti-TGF-β_1_ antibody augments BON-1 cell growth suggesting that the low proliferative index of pNET cells could be in part mediated by paracrine and autocrine TGF-β_1_ signaling [274]. Shattuck et al., observed LOH of the *SMAD3* gene in 20% of patient pNETs although no *SMAD3* inactivating mutations were found in those tumors [275]. *SMAD4*/*DPC4*, a putative tumor suppressor in pancreatic carcinoma, was mutated or deleted in 55% (5 of 9) of non-functioning pNETs but remained intact in 100% of the less aggressive, functional pNETs (insulinomas, gastrinomas, and VIPnomas) [276]. Notably, SMAD7, an inhibitor of SMAD2/3 and thus TGF-β_1_ signaling, promotes islet β cell proliferation in adult mice, supporting the tumor suppressive role of TGF-β/Smad signaling in pNETs [322].

A functional interplay between TGF-β signaling and other pNET regulators has been seen. Kaji et al., showed that menin promotes TGF-β-induced growth inhibitory and transcriptional activity by directly interacting with SMAD3 and inducing SMAD3 translocation at specific transcriptional regulatory sites [42]. This observation supports the attenuation of TGF-β/SMAD3 signaling as one of the potential mechanisms leading to multi-organ tumorigenesis in MEN1 syndrome. Leu et al., on the other hand, revealed that TGF-β inhibits BON-1 pNET cell proliferation by inducing somatostatin (SST) upregulation and secretion, thus activating the growth inhibitive autocrine loop of SST [231].

(v) Underexplored areas

Recent studies have examined the significance of non-coding RNAs, microRNAs (miRNAs), and long-non-coding mRNAs (lncRNAs), in pNET pathogenesis. miRNAs are a class of non-coding RNAs that regulate gene expression via mRNA degradation and translational repression. They often serve as biomarkers in specific cancers due to their abundance, cell-type and disease stage specificity and stability [332]. A focused analysis of tumor miRNA expression profiles in GEP-NETs (pancreatic, ileal, rectal, and appendiceal) revealed elevated levels of miR-375 across all samples [332]. Distinct NET types were able to be classified with high accuracy based on differential miRNA expression, and low-versus intermediate-grade pNETs could be discriminated from each other based on varied expression of miR-328. Examining the biological roles of these miRNAs in pNET pathogenesis is an exciting area that warrants further investigation. 

A few studies have examined circulating miRNA levels. Hellberg et al., found that NET patients displayed significantly reduced levels of miR-223 in their serum compared to healthy individuals, but found no associations between miR-223 expression and clinicopathologic characteristics of the samples [333]. Another study evaluated circulating levels of miR-29b in 45 patients with NETs (versus 19 healthy controls) and likewise observed downregulation of the miRNA in NET patient serum [334]. Similarly, miR-29b serum levels did not correlate with tumor stage or clinical features, such as tumor relapse or overall survival. These studies suggest that serum levels of miR-223 and miR-29b may be useful diagnostic biomarkers of NET but lack prognostic value.

Although initially ignored as ‘transcriptional noise’ lacking any biological function, lncRNAs have now been shown to interact with DNA, RNA, and transcription factors. In so doing, lncRNAs regulate various biological processes including DNA methylation, histone modification, and chromatin remodeling [335]. Modali et al., showed that lncRNA Meg3 has tumor-suppressive activity in pNET cells [336]. They found that Meg 3 is activated by menin and its expression is lower in mouse or human MEN1-associated pNETs compared to the normal islets. Functionally, Meg3 overexpression in insulinoma cells significantly reduces their proliferation, migration, and invasion by downregulating the c-Met (hepatocyte growth factor receptor) oncogene. A microarray-based study of patient tumors identified 280 lncRNAs that were differentially expressed in pNETs compared to the adjacent normal tissues. Subsequent pathway analyses associated these differences with multiple tumorigenic processes and signaling pathways, such as cell projection morphogenesis, cell adhesion, PI3K-Akt signaling pathway and Ras signaling [337].

pNETs have low mutational burden and as such, are traditionally perceived to be ‘cold’ tumors offering limited scope for immunotherapy. Notably, a recent study by Yuan et al., demonstrates that B7x, an immune-checkpoint ligand expressed by tumor cells, suppresses anti-tumor immunity in pNETs and as such, could be an attractive target for (p)NET immunotherapy [78]. The authors demonstrated that genetic ablation or administration of B7x antibody in *Men1*-deficient mice with insulinomas results in reduced islet cell proliferation and pNET development accompanied by increased T-cell infiltration (Table 2). This study encourages further investigation into mechanisms and approaches that will modulate immune cell activity and infiltration in pNETs.

## 3. In Vitro and Vivo pNET Models

### 3.1. Human pNET Cell Lines

Cell lines are extensively used in cancer research to better understand the underlying molecular mechanisms of tumorigenesis and facilitate identification of novel drug targets. In cancer drug discovery, cell cultures are used to screen large pools of molecules to select the most promising ones for in vivo testing.

Despite numerous efforts, only 3 pNET cell lines have been established and authenticated to date (Table 11). These pNET cell lines include QGP-1, BON-1, and NT-3. They were generated from patients with metastatic tumors and have been shown to recapitulate the parent tumors when transplanted into mice to form xenografts. The first developed and most commonly used pNET cell lines are QGP-1 and BON-1. QGP-1 was derived from a non-functional tumor in the tail of the pancreas and secretes carcinoembryonic antigen (CEA) like the parent tumor [338]. BON-1 represents a non-functional pNET and comes from a peripancreatic lymph node metastasis from a patient [339]. NT-3 is a human insulinoma cell line established from a lymph node metastasis that proliferates very slowly and exhibits a well-differentiated phenotype [340].

Genetic profiling revealed both BON-1 and QGP-1 harbor homozygous *TP53* mutations with possible loss of function [341]. That is consistent with those lines being derived from advanced metastatic pNETs, which in patients often display *TP53* and *RB1* mutations [11]. Notably, mechanistic studies using those cell lines have shown that both express functional RB1 protein and that p53 has wild-type transcriptional activity in BON-1 cells [130]. By comparison, QGP-1 are null for p53 protein [92,130]. Evidence for other pNET genetic mutations in those two lines are also somewhat conflicting. Vandamme et al., detected mutations in *ATRX* in both BON-1 and QGP-1, *TSC2* and *NRAS* in BON-1, and *KRAS* in QGP-1 [341]. However, none of these pNET genetic signatures were found by Boora et al., in their whole exome sequencing of BON-1 and QGP-1, which prompted them to question usage of these cells lines as pNET models [342]. These differences may reflect analyses of different subclones of the lines, arising through extensive passaging in culture and distribution across labs, yielding apparent differences from the parental lines or from one research group to another. The most definitive study of GEPNET cell lines was conducted by Hofving et al., in 2018 [301]. Using immunophenotyping, copy number profiling, whole-exome sequencing and drug screening, BON-1 and QGP-1 were validated as being authentic pancreatic NET cells. NT-3 cells were not evaluated in the Hofving study but were separately characterized and shown to lack mutations in *TP53, RAS,* or other genes associated with high grade neuroendocrine carcinoma [340]. However, targeted sequencing of some pNET genes revealed a homozygous missense mutation of *MEN1*.

Molecular characteristics of cell lines guide their application in specific drug testing. BON-1 cells have activated Akt-mTOR signaling and as such are a good preclinical model to test mTOR pathway inhibitors. Indeed, everolimus potently inhibited BON-1 cell growth in a dose-dependent manner, providing a strong basis for a clinical trial testing it in patients with aberrant Akt-mTOR signaling [98]. Moreover, both BON-1 and NT-3 express somatostatin receptors and have been demonstrated to be highly sensitive to somatostatin analogues [339,340]. Recently, luciferase-expressing derivatives of BON-1 and QGP-1 cells were developed to enable bioluminescent imaging of pNET metastasis in vivo [343].

The lack of pNET cell lines representing all different pNET types reflects the challenge in deriving stable neuroendocrine cell lines from patient samples. Exceptionally slow growth, fastidious nutrient conditions for survival, poor adherence to substrate, and interference by fibroblasts are some of the difficulties in deriving a pNET cell line. Nevertheless, there is an obvious need in the field to establish more diverse pNET cell lines, particularly those representing low grade, well-differentiated non-functional pNETs since those are the majority of tumors seen in patients.

### 3.2. 3D Cultures

Three-dimensional (3D) tissue culture models, organoids and spheroids, are believed to preserve the in vivo tumor architecture and microenvironment to some degree (Table 12). As such, they are sometimes preferred over cell cultures for drug screening. Wong et al., demonstrated a method to form 3D spheroids from BON-1 and QGP-1 cell lines for the first time. These cells start forming spheroids on the 3rd day of plating on an agarose-coated plate under low agitation conditions. Immunohistochemistry of spheroid sections reveal the pNET spheroids contain a highly proliferative periphery and apoptotic core, moreover, drug testing results were found to be reproducible [344]. Ear et al., established a protocol to generate SBNET spheroid cultures in extracellular matrix using surgically removed tumors [345]. Moreover, they successfully employed their SBNET spheroid model to test the cytotoxicity of rapamycin [345]. Spheroids from pNETs have also been successfully established as 3-D cultures and used for drug testing against sunitinib, everolimus, and temozolomide [346].

### 3.3. Patient Derived Xenografts

Patient-derived xenograft (PDX) tumors are increasingly being developed and used as the most authentic models of human cancer since they have been shown to faithfully replicate disease complexity [347,348]. They are developed from the transplantation of fresh or cryopreserved human tumor samples directly into an immunocompromised mouse [349,350]. Unlike xenografts derived from the pre-existing cell lines, PDXs are directly obtained from the primary patient tumor or cells without intermediate in vitro processing steps and they recapitulate the genetic and histological make-up of the original patient tumor. Since PDXs replicate the disease with high fidelity, they are good preclinical models for understanding the disease and conducting drug screenings. High throughput drug screenings performed on PDX models are felt to have higher reproducibility and translational potential compared to other pre-clinical cancer models [351].

Nevertheless, PDX models for pNETs or NETs in general are almost non-existent, despite wide-ranging attempts to generate them from different groups for over thirty years. The first reported pancreatic PDX model was developed in 2016 (Table 13) [203]. The authors subcutaneously transplanted tumor fragments from a pNET patient’s lymph node metastasis into NOD scid gamma (NSG) mice, which formed PDX tumors with an average of 80% engraftment efficiency. The successful implantation required exogenous activation of MET in the tumor cells by continued administration of a humanized antibody agonist of the receptor. The group also tried to generate PDXs from an additional 39 well-differentiated, WHO grade 1 and 2 patient pNETs, but they were only successful with one. That illustrates the remarkably challenging and unpredictable nature of the task. Nevertheless, PDXs in this study were not validated for their genetic resemblance to the primary patient tumor and no follow-up studies utilizing this model have been reported. It is possible that propagation of the PDXs over time and maintenance of the original phenotype were difficult to maintain.

Chamberlain et al., developed the first validated PDX model in 2018 (Table 13) [102]. Tumor sections removed during resection of pNET liver metastases were implanted subcutaneously into female athymic nude mice to establish the xenograft passage 0 (P0), which were subsequently passaged to obtain P1 and P2 xenografts. These PDX-pNETs were well-differentiated and retained the common pNET gene signatures, including *MEN1* loss and mTOR activation. Two different mTOR inhibitors, everolimus (mTORC1 inhibitor) and sapanisertib (a dual mTORC1 and mTORC2 inhibitor), displayed excellent antitumor activity in the PDXs although resistance to everolimus emerged in some of the tumors. Sapanisertib treatment was remarkably able to cause tumor regression in most of the everolimus resistant lesions, supporting the clinical potential of this investigational drug.

Several groups have generated PDXs from neuroendocrine neoplasm of other organs. Tanaka et al., obtained PDXs from metastatic inguinal lymph node of renal neuroendocrine carcinoma [352], whereas another group derived a ‘GA0087’ PDX model from a human gastric neuroendocrine carcinoma [353]. More comprehensive PDX generation attempts were made by Yang et al., from 106 NETs comprising 58 pancreatic, 1 gallbladder, 38 small bowel, 3 renal, and 6 liver metastases of unknown primary site [354]. Although seven of these tumors (three pancreatic, three intestinal, and one gall bladder) were successfully engrafted and gave rise to first generation xenografts, only the gallbladder PDX was sustained when re-engrafted into a new set of animals. The gallbladder PDX was propagated for eight passages without losing key molecular signatures of the original primary tumor.

Interestingly, PDXs from other NET types (e.g., pituitary) have been successfully generated in zebrafish [355,356]. In such models, stained primary cells or tumorspheres generated from post-surgical patient NETs are injected into the sub-peridermal cavity of zebrafish embryos [357,358]. This facilitates rapid evaluation of individual tumor characteristics including angiogenesis, invasion, and metastasis as well as development of precision medicine [356,358]. Some advantages of zebrafish over murine PDX models include high transplantation efficiency, limited sample size requirement, simplicity, and low cost. Moreover, the zebrafish adult pancreas shares anatomic and metabolic similarities with mammalian pancreas. However, the wide application of zebrafish PDXs could be limited by the fact that it is a non-mammalian system that requires unique expertise and facilities.

### 3.4. Genetically Engineered Mouse Models

In cancer research, genetically engineered mouse models (GEMMs) are used to understand the role of specific gene mutations in tumor development and progression in vivo. These also enable scientists to evaluate the role of tumor vasculature, tumor microenvironment, and immune system which are overlooked in in vitro systems. Moreover, GEMMS are essential tools for preclinical assessment of drug efficacy and safety, and for diagnostic or prognostic marker discovery [12].

The RIP-Tag2 (RT2) mouse is the first developed pNET GEMM (Table 2) [57]. In this pNET model, overexpression of transgenic simian virus (SV) 40 large T-antigen by rat insulin gene promoter transforms pancreatic β-cells forming hyperplastic islets by 3–5 weeks, which can further grow into angiogenic islets by 5–9 weeks and tumors by 9–12 weeks [359]. Histopathological analysis revealed that the majority of tumors in RIP-Tag2 mice are well differentiated insulinomas while a small percentage are poorly differentiated invasive carcinomas with high mitotic index resembling poorly differentiated human pNECs [360]. The latter extreme pNET phenotype is unsurprising because tumorigenesis in the RT2 model is driven by inactivation of p53 and Rb, whose genetic inactivation is more specific to high-grade, poorly differentiated pNECs in humans. Because the genetic background of this mouse model resembles that of rare pancreatic neuroendocrine carcinomas, and additionally, tumor formation is exceptionally rapid, critics of this mouse model advise its use with caution. Nevertheless, RT2 is a validated model for pNET drug screening as it was originally used in the preclinical assessment of everolimus and sunitinib. Most impressively, their results successfully predicted the outcomes of clinical trials [95,361], leading to the use of those agents in patient therapies today.

The genetic background of RIP-Tag2 mice influences the type of pNET they develop. Under the original C57BL6 background, RIP-Tag2 mice form insulinomas. By comparison, RIP-Tag2; AB6 F1 hybrid mice generated by breeding RIP-Tag2 (C57BL6) males with wild type A/J strain females switch to developing non-functional pNETs in a high percentage of animals [58]. This is the first model of non-functional tumors, the most common pNET in patients, making it a relevant and powerful model for future investigations. Genetic studies reveal RT2 AB6F1 mice inherently express reduced levels of Insm1, a β-cell specific transcription factor required for insulin synthesis compared to their C57BL6 counterparts. RIP-Tag2; AB6 F1 mice also exhibited higher frequency of liver metastases, which is consistent with findings that loss of the *Insm1* gene enhances stem cell-like properties, invasiveness and metastatic potential of the pNET cells [58].

Many mouse models of human MEN1 syndrome or somatic *Men1* loss associated with sporadic pNETs have been developed, and all result in insulinomas. Homozygous deletion of *Men1* is embryonically lethal in mice. Death occurs in utero between 11.5 and 12.5 embryonic days due to multiple developmental disorders that include cardiac hypertrophy, non-closure of the neural tube, and defects in liver anatomy [61,62]. Heterozygous *Men1* mutation, on the other hand, recapitulates the autosomal dominant nature of familial MEN1 syndrome. Animals that are *Men1*^+/−^ develop tumors with metastatic potential in the endocrine pancreas, pituitary, parathyroid, thyroid, and adrenal glands in a multistage fashion mimicking the human disease [61,62]. Somatic mutations in *MEN1* are strongly associated with sporadic pNET genesis [23,24].

To overcome the embryonic lethality of homozygous *Men1* knockout, conditional deletion models have been developed to specifically assess the role of menin in the developing mouse pancreas and pNET progression. Mice expressing transgenic Cre recombinase under the rat insulin promoter (RIP) were crossed with *Men1*-floxed mice to delete *Men1* in pancreatic β-cells. These mice developed β-cell hyperplasia in 2–4 months and insulinomas starting at 6–9 months [72,76,77]. Mice with homozygotic loss of *Men1* in β-cells exhibited earlier incidence of insulinomas than the heterozygotes [67]. Importantly, RIP-associated Cre expression is not confined to pancreatic β-cells and also occurs in pituitary glands, resulting in *Men1* loss and formation of pituitary prolactinomas [65,72,76]. This is not a setback in the study of *Men1* loss in mice as patients of MEN1 syndrome also develop pituitary tumors in addition to pNETs.

Tumor formation in *Men1* mouse models is delayed even with biallelic loss of *Men1* in β-cells, making these animals less than optimal for drug efficacy testing. Prolonged tumor latency in these mice suggests that additional genetic alterations cooperate with *Men1* loss to drive pNET development [67]. Indeed, Wong et al., showed that combined loss of *Pten* and *Men1* induces pNET development with shorter latency compared to loss of either gene alone, underscoring the cooperativity of these two tumor suppressors. The same study also compared the use of RIP vs. mouse insulin promoter (MIP) for Cre expression in their conditional knockout models. While RIP Cre; *Men1^flox^*^/*flox*^ *Pten^flox^*^/*flox*^ mice developed pancreatic and pituitary NETs, as previously demonstrated by other groups, MIP Cre; *Men1^flox^*^/*flox*^ *Pten^flox^*^/*flox*^ mice developed only pNETs, indicating MIP more accurately provides β-cell specificity [56].

Various groups have attempted to model glucagonomas by inducing pancreatic α-cell hyperproliferation via genetic manipulations in mice [69,70,71,72]. Hanahan et al., the group that generated RT2 mice, also developed Glu-Tag mice expressing SV40 large T-antigen under the preproglucagon promoter to induce glucagonomas [70]. Lu et al., (2010) used Cre recombinase expressed under the proglucagon promoter to delete *Men1* in pancreatic α-cells. Although glucagonomas were witnessed by 6 months, the majority of pNETs in these mice were insulinomas. Genetic cell lineage tracing revealed the α-cell lacking *Men1* can transdifferentiate into insulinomas, suggesting *Men1* could be a regulator of α-cell plasticity [69]. Other approaches for modeling glucagonoma in the mouse include enhancing pancreatic α-cell proliferation by antagonizing glucagon signaling [71] or by inhibiting an enzyme (e.g., proconvertase 2) required for glucagon synthesis [72]. Interestingly, these mice also develop mixed islet tumors in addition to expected glucagonomas, supporting the idea that hyperplastic α-cells can transdifferentiate into tumors of other islet cell types or that hyperplastic α-cells simply cross-talk with other islet cells inducing their hyperproliferation.

To determine the role that *Cdkn2a* (or *Ink4a*-*Arf*) and *p53* loss play in pNET genesis, Azzopardi et al., programmed conditional loss of either or both tumor suppressor loci in pancreatic progenitor cells together with RIP-mediated overexpression of oncogenic polyoma middle T antigen (PyMT) [73]. Concomitant loss of both *Ink4a-Arf* and *p53* led to a higher incidence (40%) of pNETs compared to loss of either locus alone (20% for *Ink4a-Arf* versus 17% for *p53*). The usefulness of this model is compromised by inconsistency in pNET development and involvement of PyMT co-expression, which activates several pathways including MAPK, PI3K, and Hippo signaling.

In sum, there remain a relatively limited number of pNET cell and mouse models available for investigators to study pNET biology and therapy. While existing GEMMs of the disease are extremely valuable, more mouse models of non-functional pNETs are urgently needed since those represent the majority of pNETs seen in patients. Recent work by the Bibb group may provide an invaluable, inducible mouse model of both functional and non-functional pNETs. Their GEMM employs doxycycline controlled hyperactivation of CDK5 in pancreatic beta cells [362], reflecting their earlier finding that all types of human NETs, including pNETs, overexpress hyperactive CDK5 [363]. Indeed, greater insights into essential mechanisms driving pNETs, from analyses of human tumors and cell lines, are expected to guide and in turn be facilitated by the development of new GEMMs. Progress in both areas should advance preclinical assays of novel therapeutics. In that regard, more progress in establishing patient-derived spheroid, organoid, and xenograft models will likewise provide additional, powerful systems for translational NET research moving forward.

## 4. Concluding Thoughts

Most pNETs are sporadic, non-functional tumors that too often remain undiagnosed until their advanced, metastatic stages. Although current therapies targeting mTOR, RTKs and SSTs effectively increase patient survival, new treatments are urgently needed for tumors that are unresponsive or have become resistant to those systemic treatments. A deeper understanding of proteomic changes and mechanisms driving pNET molecular pathogenesis will help identify new targets for therapies that will circumvent pNET resistance.

This review describes many of the genes and pathways frequently dysregulated in pNETs, both inherited and sporadic, while highlighting the fact that how those alterations facilitate tumorigenesis remains only partly understood. Addressing this gap in knowledge will be critical to the development of new targeted therapies. In that regard, the field has little information about the pNET metabolome and proteome. Moving forward, global metabolomic, proteomic, and phosphoproteomic studies of patient pNETs, cell lines, and mouse tumor models paired with functional studies of significantly altered metabolites and proteins are expected to define meaningful molecular events driving the disease. A recent study of phosphoprotein-based biomarkers in medullary thyroid carcinoma (MTC) NETs by the Bibb group revealed the value of analyzing post-translational modifications to assess the signaling status of tumors [364]. Such information should not only guide new therapies but also add depth to genetics-based tumor classifications that will better stratify patients for optimal clinical management.

Numerous studies have shown various pNET pathways collaborate to drive the tumor development and progression. These cooperative molecular interactions can be capitalized upon to develop novel combination therapies. Along those lines, identification of master regulatory proteins or events governing important pNET-associated pathways may enable simultaneous targeting of multiple pathways using fewer drugs. This could reduce drug induced toxicities and potentially avoid the emergence of resistant tumors.

Importantly, continued efforts are needed to develop new pNET models, particularly for the more common, non-functional pNETs. Such advances in building more cell lines, patient-derived spheroids/organoids, and in vivo models that accurately recapitulate the indolent nature of the disease will greatly accelerate pNET research and therapy.

## Figures and Tables

**Table 1:** Molecular profiling studies revealing MEN1 alterations in pNETs.

Technique	Reference	Key Findings
Exome/genome sequencing	[24]	Somatic *MEN1* mutations in 44.1% of 68 sporadic pNETs. *MEN1* mutations correlated with poor patient survival.
	[23]	Somatic *MEN1* mutations in 41% of 102 primary pNETs. Abnormal telomere length observed in *MEN1*-mutated tumors.
	[39]	In total, 26% of 57 sporadic well differentiated pNETs had recurrent LOH of 10 specific chromosomes and biallelic *MEN1* inactivation. Another 40% had chromosome 11 LOH and biallelic *MEN1* inactivation. The first patient group had worse clinical outcomes compared to the second.
	[40]	*MEN1* mutations in 43% of 65 pNETs.
	[43]	Somatic *MEN1* mutations in 56% of 80 patient pNETs. In total, 1 of 17 patients carried a germline *MEN1* mutation.
Allelotyping/LOH analysis	[31]	Loss of chromosomal segment 11q (where *MEN1* is located) in >60% cases.
	[34]	LOH of 11q13 in 70% and *MEN1* mutations in 27% of 11 advanced pNETs (9 NF and 2 glucagonomas).
	[29]	*MEN1* mutatons in 6 (mostly pNETs) of 43 sporadic GEPNETs.
	[37]	*MEN1* allelic deletions in 93% of gastrinomas and 50% of 12 insulinomas; mutations in 33% gastrinomas and 17% insulinomas.
Microarray	[35]	Consensus cluster analysis of microarray results showed clustering of 5 out of 9 sporadic NF-pNETs with MEN1-associated familial pNETs. In total, 4 of those 5 sporadic pNETs had *MEN1* LOH.

**Table 2:** Genetically engineered pNET mouse models.

Model	Strain	pNET Type	Key Findings
RIP-Tag2	C57BL/6	Insulinoma	SV40 large T-antigen inactivates p53 and Rb in islet-β cells and promotes insulinoma development in a multi-stage, synchronized fashion [57]
*Men1*^f/f^ *Pten*^f/f^; MIP-Cre or RIP-Cre (MPM or MPR)	C57BL/6J	Insulinoma	Loss of *Pten* co-operates with that of *Men1* to develop well differentiated G1/G2 pNETs [56]
RIP-Tag2 AB6F1	AB6F1 (hybrids from A/J dam and RT2 C57BL/6 sire)	Non-functional (NF) pNETs	RT2 mice develop NF pNETs with higher rate of liver metastases on AB6F1 genetic background, which is attributed to low expression of Insm1, a β-cell specific differentiation factor required for insulin secretion [58]
RIP-MyrAkt1	C57/BL6	Insulinoma	β-cell specific expression of constitutively active Myr-Akt leads to formation of insulinomas in S6K1 (a mTOR downstream target) dependent manner [59]
Avp-Tag	C57B1/10; CBA/J	Insulinoma	Mice bearing vasopressin promoter (1.2 kb 5′ sequence)-SV40 hybrid transgene uncharacteristically transformed pancreatic β-cells and anterior pituitary cells with no effect in hypothalamus and other organs where vasopressin is normally expressed [60]
*Men1* ^TSM/+^	NIH Black Swiss; 129/Sv	Insulinoma	Mutation of one *Men1* allele by homologous recombination leads to insulinoma development by 9 months and other tumors involving parathyroid, thyroid, adrenal cortex, and pituitary by 16 months mimicking human MEN1 syndrome [61]
*Men1* ^+/T^	129	Insulinoma and glucagonoma that dedifferentiated into advanced NF-pNETs	Disruption of one *Men1* allele by gene targeting resulted in tumors of pancreatic, parathyroid, thyroid, pituitary, and adrenal glands exhibiting multistage progression and metastatic potential [62]
*Men1*^f/f^; RIP-Cre	C57BL/6	Insulinoma	RIP-Cre mediated conditional knockout of *Men1* gene leads to insulinomas and pituitary prolactinomas by 9 months [63]
*Men1*^f/f^; Glu-Cre	Not specified	Insulinoma	Surprisingly, loss of *Men1* in α-cells resulted in β-cell insulinomas rather than glucagonomas suggesting the role of intercellular talk between islet cells [64]
*Men1*^f/f^; Pdx1-Cre	FVB; 129Sv	Insulinoma	Although *Men1* is lost in both pancreatic exocrine and endocrine cells, only endocrine cells developed into highly angiogenic tumors suggesting the role of tissue-specific menin modulators and surrounding microenvironment during tumorigenesis [65]
*Men1*^f/f^; RIP2-CreER	129; (C57BL/6 X CBA)	Insulinoma	Temporally controlled β-cell specific loss of *Men1* led to insulinomas. Moreover, the model helps elucidate early stage events such as β-cell hyperproliferation [66].
*Men1*^f/f^; RIP-Cre	B6; FVB; 129Sv	Insulinoma	Conditional knockout of both *Men1* alleles promoted islet cell tumor development much faster than that of one allele [67]
*Men1*^f/f^; RIP-Cre	129	Insulinoma	Disruption of *Men1* gene directly in β-cells led to insulinoma development by 6 months in a multistage fashion, exhibiting angiogenesis and altered E-cadherin and β-catenin expression [68]
*Men1*^f/f^; Glu-Cre	129; B6/CBAJ-F1	Insulinoma, glucagonoma, and mixed islet cell tumor	α-cell specific loss of *Men1* leads to α-cell hyperplasia that grow into glucagonomas, however, majority of the hyperplastic α-cells transdifferentiate into insulinomas and mixed islet tumors [69]
Glu2-Tag	C57BL/6	Glucagonoma	Expression of Tag under preproglucagon promoter drives hyperproliferation of alpha cells and formation of glucagonomas by 9–12 months. Promiscuous expression of T-antigen in hind brain neurons is not sufficient for their hyperplasia or tumorigenesis [70].
*Gcgr* ^−/−^	DBA/1	Glucagonoma	Inhibition of glucagon signaling by glucagon receptor mutation causes α-cell hyperplasia that progress into islet dysplasia and solid tumors. A few animals develop mixed tumors or NF-pNETs [71].
*Pc2* ^−/−^	C57BL/6	Glucagonoma	Loss of prohormone convertase 2 required for glucagon synthesis leads to α-cell hyperplasia that develop glucagonomas and mixed islet tumors by 6–8 months [72]
RIP7-rtTA; tet-o-MT; p48-Cre; *Ink4a*/*Arf* ^f/f^	C57BL6; FVB; ICR	Not determined	Loss of *Ink4a*/*Arf* tumor suppressor locus cooperates with overexpression of PyMT in pancreatic progenitor cells to induce pNET formation however at low incidence rate of 20% [73]
RIP7-rtTA; tet-o-MT; p48-Cre; *Trp53*^f/f^; *Ink4a*/*Arf* ^f/f^	C57BL6; FVB; ICR	Not determined	Overexpression of oncogenic PyMT in β-cells together with deletion of *P53* and *Ink4a*/*Arf* loci results in pNET incidence in 40% mutant mice [73]
pIns-c-MycER^TAM^/RIP-*Bcl-x_L_*	CBAxC57BL/6	Insulinoma	Conditional expression of transgenic *Myc* and *Bcl-x_L_* to suppress Myc-induced apoptosis in islet β cells causes islet tumor development in a reversible fashion [74]
Pdx1-Cre; *Trp53*^R172H^;*Rb*^f/f^	FVB/N; J1;	Well differentiated, metastatic insulinoma and glucagonoma	Pancreas-specific *p53* mutation and *Rb* deletion caused islet dysplasia that progressed to indolent and metastatic pNET in stepwise fashion [75]
*Men1*^f/f^ *Rb1*^f/f^ RIP-Cre; *Men1*^f/f^ Pten^f/f^ RIP-Cre; *Trp53*^f/f^ *Rb1*^f/f^ RIP-Cre;	*Men1*^f/f^ (129S, FVB) *Rb1*^f/f^(FVB;129) Pten^f/f^(C;129S4) Trp53^f/f^(B6.129P2) RIP-Cre (C57BL/6)	Well differentiated G1, G2, and G3 pNETs (insulinoma)	Demonstrated the cooperative role of tumor suppressor genes, *Men1*, *Rb1*, *Pten*, and *Trp53* in pNET suppression [76]
RIP-Tag2; *Rabl6*^m/m^	C57BL/6N	Insulinoma	Loss of oncogenic RABL6A attenuates pNET progression and angiogenesis in RIP-Tag2 mice [77]
Pdx1-Cre; *Men1*^f/f^; B7x KO	C57BL/6	Insulinoma	Loss of B7x, an immune-checkpoint ligand, reduces islet β-cell proliferation and pNET formation consistent with increased T-cell infiltration [78]

(Abbreviations: RIP = rat insulin promoter, MIP = mouse insulin promoter, Tag = T-antigen).

**Table 3:** Molecular profiling studies revealing PI3K-Akt-mTOR signaling pathway alterations in pNETs.

Technique	Reference	Key Findings
Exome sequencing	[24]	Somatic *PTEN*, *TSC2*, and *PIK3CA* mutations in 7.3%, 8.8%, and 1.4% of 68 sporadic pNETs, respectively.
	[23]	Somatic mutations in mTOR pathway genes observed in 102 primary pNETs: *PTEN* (7%), *DEPDC* (2%), *TSC1* (2%), and *TSC2* (2%). mTOR pathway gene mutations associated with poor survival.
	[40]	11% of 65 pNETs had mTOR pathway gene mutations: *TSC2* (6%) and *PTEN* (5%).
	[43]	*TSC2* mutations in 25% of 80 patient pNETs. In total, 1 of 17 patients carried a germline TSC2 mutation.
Allelotyping/LOH analysis	[87]	LOH of 10q23 (where *PTEN* is located) in >50% of 22 pNETs. *PTEN* mutations rarely observed.
	[86]	Allelic deletions of 16p13 (where *TSC2* is located) in 36% of 28 pNETs
Microarray	[88]	*TSC2, PTEN* or both downregulated in 85% of primary PNETs, subsequently validated by qRT-PCR and IHC studies. Reduced expression of *TSC2* and *PTEN* correlated with poor patient prognosis.
RNA sequencing	[106]	Ingenuity pathway analysis (IPA) and Connectivity Map (CMap) analysis of 626 metastatic gene signatures obtained from 39 primary tumors, 21 lymph node metastases and 17 liver metastases predicted mTOR and PI3K as top pNET pharmacological targets.
	[92]	Alteration of Akt signaling genes revealed by sequencing of 20 primary pNETs.
IHC	[107]	Low PTEN expression in 48% of 21 pNETs.

**Table 4:** Molecular profiling studies revealing INK4a/ARF and RB1 pathway alterations in pNETs.

Technique	Reference	Key Findings
Sequencing and mutational analysis	[24]	No *INK4A*/*ARF* mutation observed in 68 pNETs.
	[11]	Inactivating mutations in the *RB1* gene identified in 75% (3/4) small cell pNECs and 66.67% (2 of 3) large cell pNECs, however, absent in 11 well-differentiated pNETs analyzed. No *CDKN2A* mutations observed in 7 pNECs and 11 pNETs.
Methylation Specific PCR (MSP)	[112]	In total, 91.7% of 12 gastrinomas and non-functioning pNETs demonstrated *INK4a* homozygous gene deletions (41.7%) or 5′ CpG island hypermethylation. However, no mutations were found by single-strand conformation polymorphism (SSCP) analyses.
	[103]	*INK4a* 5′-CpG island hypermethylation in 52% of 44 gastrinomas, along with homozygous gene deletions.
	[126]	In total, 17% of 17 insulinomas exhibited *INK4a* gene alterations: homozygous deletion in 5.9% and promoter hypermethylation in 11.8%. No *INK4a* mutations were identified by SSCP. IHC confirmed loss of the p16 protein expression in samples harboring gene alterations.
	[116]	*INK4a* CpG island hypermethylation in 17% of 12 pNETs, more frequently in malignant (29%) than in benign (0%) tumors.
PCR (MSP or gene specific and LOH)	[114]	*INK4a* and *ARF* CpG island hypermethylation in 9% (1 out of 11) pNETs each vs. 44% and 31%, respectively, in carcinoid NETs. Chromosome 9p loss identified in 18% (2 of 11) pNETs.
RT-PCR	[104]	Absent expression of *INK4a*, *INK4b*, and *ARF* in 28% (2/7), 57% (4/7), and 43% (3/7) NF-pNETs. Loss of *INK4b* observed in 26% insulinomas and gastrinomas (N = 19), however, *INK4a* and *ARF* found to be expressed.
	[35]	Overexpression of CDK4 and CDK6 in MEN1 NF-pNETs (N = 10) compared to VHL (N = 9) and sporadic (N = 9) NF-pNETs and normal islets (N = 4).
Tissue microarray, qPCR, and FISH	[122]	IHC revealed high CDK4, cyclin D1 and phospho-RB1 levels in 58–68% of total pNETs (N = 92) in contrast to negative staining in the normal pancreas. qRT-PCR revealed marked upregulation of *CDK4* in 19% of 26 pNETs, which were found to have amplified *CDK4* or *CDK6* genes by qPCR and FISH.
IHC	[11]	RB1 protein expression intact in well-differentiated pNETs, however, lost in 88.9% of 9 small cell and 60% of large cell pNECs. Loss of p16 expression observed in pNECs with intact RB1 suggest p16/Rb pathway is disrupted in virtually all pNECs.

**Table 5:** Molecular profiling studies revealing ATRX/DAXX alterations in pNETs.

Technique	Reference	Key Findings
Exome sequencing and mutational analysis	[24]	Somatic *ATRX* and *DAXX* inactivating-to-missense mutations in 25% and 17.6% of 68 sporadic pNETs, respectively. IHC showed complete loss of *ATRX* or *DAXX* in pNETs harboring the corresponding gene mutations. *ATRX*/*DAXX* mutations with or without *MEN1* mutations correlated with improved patient survival.
	[23]	Somatic mutations in *ATRX* (10%) and *DAXX* (22%) of 102 primary pNETs. *ATRX*/*DAXX* mutations linked with longer telomere length and poor patient survival.
	[40]	*DAXX* and *ATRX* mutations in 28% and 11% of 65 pNETs, respectively. These mutations were mutually exclusive.
	[43]	Somatic alterations in *DAXX* and *ATRX* observed in 40% and 25% of 80 patient pNETs.
IHC	[11]	ATRX or DAXX immunolabeling lost in 45% of 11 pNETs, but intact in all of 19 pNECs.

**Table 6:** Molecular profiling studies revealing p53 pathway alterations in pNETs.

Technique	Reference	Key Findings
Sequencing and mutational analysis	[11]	Inactivating *TP53* mutations found in 4 of 7 pNECs, however, in none of 11 well-differentiated pNETs.
	[24]	*TP53* gene mutations identified in 3% of 68 pNETs.
PCR and IHC	[157]	Amplification of the *MDM2* gene in 22% (38 of 169), *MDM4* in 30% (45 of 150), and *WIP1* in 76% (34 of 45) well differential pNETs, which correlated with corresponding changes in mRNA and protein expression.
IHC	[11]	11 well differentiated pNETs exhibited normal p53 labeling, however, abnormal labeling observed in 9 (100%) small cell pNECs and 9 of 10 (90%) large cell pNECs.

**Table 7:** Molecular profiling studies on growth factor signaling in pNETs.

Technique	Reference	Key Findings
FISH	[185]	*EGFR* copy number found to be elevated in 38% of 44 pNET cases.
qRT-PCR	[186]	Positive *VEGF* expression in 5 of 8 pNETs and *EGFR* in 4 pNETs of which 2 had overexpressed *EGFR* compared to the normal pancreatic tissue.
IHC	[178]	Positive expression of VEGF in 16 (11 mild, 3 moderate, and 2 strong) out of 20 pNETs.
	[187]	Of 38 malignant pNETs, 100% expressed PDGFRα tumor cells, 57% in stromal cells; 74% expressed PDGFRβ in tumor cells, 97% in stromal cells; 92% of tumors expressed c-kit and 55% expressed EGFR.
	[180]	Positive VEGF-A expression in all 19 primary well-differentiated pNETs and 7 liver metastases. Mild to moderate VEGF-C immunostaining in the majority of primary pNETs with significantly increased expression in liver metastases. High immunoreactivity for VEGFR2 observed in all 8 primary pNETs and 3 liver metastases examined.
	[179]	Positive expression of VEGF in pNETs 73% (33/45) pNETs which correlated negatively with WHO disease stage. In total, 91% (41/45) pNETs showed high HIF-1α staining.
	[188]	Positive expression of EGFR or p-EGFR in 25–50% of 48 primary pNETs or their metastases. Higher percentage of carcinoid NETs were found to be EGFR- or p-EGFR positive. Activated p-EGFR in primary NETs correlated with poor prognosis.
	[189]	Positive PDGFRβ expression observed in 18 of 21 (86%) primary tumors and all of 19 pNET metastases. PDGFRβ shown to be more frequently expressed in primary pNETs and metastases as compared to normal endocrine pancreas.
	[177]	In total, 80% of 15 pNETs showed mild to moderate VEGF staining.
	[185]	High IHC staining (score 3) of VEGFR1, TGFBR1, PDGFRA, SSTR5, SSTR2A, and IGF1R in 80%, 69% 65%, 55%, 55%, and 47% of 44 pNETs, respectively.

**Table 8:** Molecular profiling studies on Ras-MAPK signaling in pNETs.

Technique	Reference	Key Findings
Sequencing and mutational analysis	[24]	*KRAS* mutation observed in none of the 68 pNETs studied.
	[155]	*HRAS*, *NRAS*, or *BRAF* mutations in less than 1% pNETs.
ctDNA sequencing	[212]	ctDNA NGS of 280 NET/NEC patient plasma samples revealed *KRAS* mutations in 22% (n = 61) and *NF1* mutations in 7% (n = 19) samples.
Mutational analysis	[185]	No pNETs (n = 35) showed *KRAS* exon 2 mutations.

**Table 9:** Molecular profiling studies on SSTR signaling in pNETs.

Technique	Reference	Key Findings
RT PCR and IHC	[230]	mRNA amplification of *SSTR1* in 90.1%, *SSTR2* in 84.8%, *SSTR3* in 78.8%, *SSTR4* in 24.2% and *SSTR5* in 42.4% of pNET cases. Positive SSTR2 immunoreactivity in 15 of 22 tumors (68.2%), SSTR3 in 8 of 22 (36.4%), and SSTR5 in 14 of 22 (63.6%).
Microarray and IHC	[88]	SSTR2 expression is significantly upregulated in NF-pNETs compared to insulinomas.
IHC	[239]	Mild to strong immunoreactivity for SSTR2A observed in 15 out of 16 (94%) pNETs.
	[238]	Positive (IHC score 1 to 4) SSTR2A immunostaining in 63% of 79 pNETs. Negative staining correlated with poor outcomes.
	[185]	Positive SSTR2A and SSTR5 staining in 68% and 58% of 44 pNETs, respectively.
	[240]	Immunoreactivity for SSTR1 in 40%, SSTR2A in 90%, SSTR2B in 39%, SSTR3 in 51% and SSTR5 in 76% of 71 NETS of the GI tract and lungs.
	[241]	Positive immunostaining of SSTR1 and SSTR2 in 100% of 11 G1 and G2 NETs of the GI tract and lungs.
	[229]	Positive SSTR2A and SSTR5 staining in 86% and 35%, respectively, of 99 pNETs. Positive SSTR2A expression correlated with better overall survival.

**Table 10:** Molecular profiling studies on miscellaneous pNET-associated proteins and pathways.

Technique	Reference	Key Findings
IHC	[107]	In total, 17 out of 21 patients (81%) pNETs showed strong immunoreactivity for Myc.
IHC	[260]	Positive Myc expression in all 39 benign or metastatic pNETs.
Microarray	[270]	Upregulation of LCK, a Src family kinase, in primary (n = 8) and metastatic pNETs (n = 5), and pNET cell lines compared to normal islets. Microarray results were validated by qRT-PCR and IHC.
qPCR and IHC	[130]	*RABL6A* amplification in 6 of 11 primary pNETs and their matched metastases. High RABL6A protein expression observed by IHC in all pNETs (n = 5).
IHC	[271]	Significant upregulation of all HDACs (I, IIa, IIIb, III and IV) in pNETs whose expression was found correlate with tumor grading and predict disease outcomes.
RNA seq	[261]	Transcriptome analysis of 212 patient GEP-NETs complemented by systematic drug perturbation assays identified HDAC class I inhibitor, entinostat, as a potent agent to treat 42% GEP-NET patients.
RNA Seq	[106]	IPA and cMAP analysis of differentially expressed genes in 43 primary pNETs vs. their matched metastases predicted HDAC as one of the top pharmacological targets to treat metastatic pNETs.
IHC	[185]	Positive immunoreactivity for HSP90 and TGF-βRI in 75% of 67 primary and metastatic pNETs.
IHC	[262]	Positive aurora kinase A expression in 8 of 10 insulinomas, in all of 13 nonfunctional pNETs and 20 SBNETs.
IHC	[272]	Ptch1, the sonic hedgehog receptor, expressed in 12 of 22 sporadic pNETs and 4 of 5 MEN-1 pNETs with no significant correlation with clinical outcomes.
IHC	[273]	Nuclear β-catenin immunostaining in higher percentage of stage III/IV pNETs (2/13, 15%) vs. stage I/II pNETs (0/74). Negative APC expression in 70% (57/81) of the cases.
IHC	[274]	Positive expression of TGF-β, TGF-βRI, and TGF-βRII in 75–100% patient pNETs.
LOH and sequencing	[275]	LOH of *Smad3* in 20% of 20 pNETs; no inactivating *Smad3* mutations observed.
PCR and SSCP mutational analysis	[276]	DPC4 mutation or deletion detected in 55% (5 of 9) non-functional pNETs in contrast to none of the 16 functional tumors-insulinomas, gastrinomas, and VIPnomas.

**Table 11:** In vitro models: cell lines.

Cell Type	Source	Key Features
QGP-1	Metastatic human islet cell carcinoma	Production of carcinoembryonic antigen and absence of hormonal secretion recapitulating the parent tumor [338].
BON-1	Peripancreatic lymph node metastasis of pancreatic carcinoid tumor	Express gastrin and somatostatin receptors; synthesize serotonin and chromogranin [339].
NT-3	Lymph node metastasis of an insulinoma	Model of well-differentiated insulinoma, highly expresses somatostatin receptors and neuroendocrine markers, exhibit slow growth in contrast to BON-1 and QGP-1 cells. Gene sequencing revealed a homozygous missense mutation in *MEN1* gene with other pNET-associated genes intact [340].

**Table 12:** In vitro models: 3D cell cultures.

Type	Source Cell/Tissue	Key Features
pNET spheroids	pNET cell lines: BON-1 and QGP-1 Human lung neuroendocrine cell line: H727	Three-dimensional spheroids start forming by day 3 of cell plating and contain highly proliferative cells at the periphery and a necrotic center, proposed to be useful models for in vitro drug screening [344].
SBNET spheroids	Patient SBNETs	Cultured with the help of extracellular matrix (Matrigel); doubling time was 14 days; expressed synaptophysin, chromogranin and SSTR2; undergo apoptosis with rapamycin treatment [345].
pNET spheroids	Patient pNETs	Primary tumor cells isolated from pNETs and cultured in vitro to form islet-like tumoroids that retain a neuroendocrine phenotype and are viable for at least 2 weeks in culture for drug testing [346].

**Table 13:** Patient-derived xenografts (PDXs).

Reference	Source Tissue	Key Features
[203]	Primary tumor fragments from patients’ lymph node metastases used for s.c. transplantion into NOD scid mouse (NSG)	Successful engraftment required MET proto-oncogene activation by HGF or its analogue. Critical comments: validity, utility unclear, no studies utilizing the model.
[102]	pNET liver metastases	pNETs were well-differentiated, pNET gene signatures were retained, tumor growth inhibition observed with everolimus and sapanisertib.
